# Fatty acid-binding protein 2 suppresses liver metastasis by cytoplasmic sequestration of β-catenin to enhance ferroptosis sensitivity in colon cancer

**DOI:** 10.1038/s41419-026-09161-7

**Published:** 2026-08-01

**Authors:** Long Wu, Huan Wu, Yu-Tong Zhou, Guang-Ling Ou, Yong Sun, Xiao-Yun Li, Yun-Huan Zhen, Kun Cao, Hai-Yang Li

**Affiliations:** 1 Department of Colorectal Cancer Center, the Affiliated Hospital of Guizhou Medical University, Guiyang, Guizhou Province China; 2 The Guizhou Provincial Key Laboratory of Digestive System Diseases, Guiyang, Guizhou Province China; 3 Department of Infectious Diseases, the Affiliated Hospital of Guizhou Medical University, Guiyang, Guizhou Province China; 4 School of Clinical Medicine, Guizhou Medical University, Guiyang, China; 5 Key Laboratory of Hepatobiliary and Pancreatic Diseases Treatment and Bioinformatics Research, Guizhou Medical University, Guiyang, China; 6 Department of Hepatobiliary Surgery, the Affiliated Hospital of Guizhou Medical University, Guiyang, Guizhou Province China

**Keywords:** Metastasis, Colon cancer

## Abstract

Metastatic colon cancer commonly exhibits resistance to ferroptosis, an iron-dependent form of regulated cell death driven by lipid peroxidation. However, the mechanisms linking metabolic regulation to ferroptosis during metastatic progression remain incompletely understood. Here, we identified fatty acid-binding protein 2 (FABP2) as a regulator associated with ferroptosis sensitivity and liver metastatic phenotypes in colon cancer. Multi-omics analyses of clinical specimens showed reduced FABP2 expression in advanced colon cancer, which was associated with metastatic progression and poor survival. Mechanistically, FABP2 interacted with β-catenin, altered its intracellular localization, and was associated with reduced β-catenin/TCF4-dependent transcription of glutathione peroxidase 4 (GPX4), a key regulator of ferroptosis resistance. FABP2 overexpression increased ferroptosis sensitivity, whereas FABP2 downregulation promoted GPX4-dependent ferroptosis evasion and metastatic phenotypes. Pharmacological inhibition of Wnt/β-catenin signaling or GPX4 activity partially attenuated the effects of FABP2 loss. Together, these findings identify FABP2 as a factor associated with ferroptosis regulation and metastatic progression in colon cancer, and provide additional insight into the interplay between lipid metabolism, Wnt/β-catenin signaling, and ferroptosis.

## Introduction

Colon cancer remains a leading cause of cancer-related mortality globally, with liver metastasis representing a major determinant of poor prognosis [1–3]. Despite advances in surgery and systemic treatment, long-term survival of patients with metastatic colon cancer remains unsatisfactory [4, 5], underscoring the need to elucidate the molecular mechanisms underlying metastatic progression.

A major challenge in metastasis research is understanding how disseminated tumor cells survive and proliferate within the microenvironment of distant organs. The liver, in particular, presents a metabolically demanding environment characterized by elevated oxidative stress and abundant iron availability [6, 7]. Successful colonization of this niche requires tumor cells to develop adaptive mechanisms that protect against oxidative stress-induced cell death [8]. Ferroptosis, an iron-dependent form of regulated cell death driven by lipid peroxidation, has emerged as an important barrier to metastatic colonization [9–11]. Increasing evidence suggests that resistance to ferroptosis is critical for the survival of metastatic tumor cells in the liver. However, the molecular mechanism governing ferroptosis evasion during metastatic progression remains incompletely understood.

In colon cancer, the Wnt/β-catenin signaling pathway is a central regulator of tumor progression and cell survival and is aberrantly activated in most cases [12, 13]. Beyond its established roles in proliferation and stemness, recent studies indicate that β-catenin contributes to ferroptosis resistance through transcriptional upregulation of glutathione peroxidase 4 (GPX4), a key enzyme that limits lipid peroxidation [14]. These findings suggest that the Wnt/β-catenin-GPX4 axis may contribute to metastatic adaptation. However, the upstream regulators that modulate this pathway during metastatic progression remain poorly defined. Given the central role of lipid metabolism in ferroptosis, we reasoned that proteins involved in intracellular lipid handling may participate in regulating this survival pathway.

Members of the fatty acid-binding protein (FABP) family are important regulators of intracellular lipid transport and metabolic signaling and have increasingly been implicated in tumor progression [15–17]. Previous studies have mainly focused on FABP4 and FABP5 [18–20], whereas the intestine-enriched isoform, FABP2, has received little attention in cancer biology. Given its tissue specificity and role in lipid metabolism, we sought to determine whether FABP2 plays an unrecognized role in colon cancer progression, particularly during liver metastasis.

In the present study, we investigated the role of FABP2 in colon cancer progression and liver metastasis, with particular focus on its involvement in ferroptosis regulation. We found that FABP2 expression was reduced in colon cancer and associated with tumor progression, metastatic phenotypes, and clinical outcome. Mechanistically, our findings support a link between FABP2 and Wnt/β-catenin signaling through altered β-catenin intracellular localization, accompanied by changes in GPX4 expression and ferroptosis-related phenotypes. Functional analyses further examined the contribution of this pathway to malignant progression and liver metastatic colonization. Together, these findings provide insight into the interplay between lipid metabolism, ferroptosis regulation, and metastatic progression in colon cancer.

## Materials and methods

### Human clinical specimens

Primary colon cancer tissues and paired adjacent non-tumor tissues were collected from patients treated at the Affiliated Hospital of Guizhou Medical University. This study was conducted in accordance with the Declaration of Helsinki and was approved by the Institutional Ethics Committee of the Affiliated Hospital of Guizhou Medical University (Approval Number: 2025LS211). Written informed consent was obtained from all participants prior to sample collection. Detailed clinical characteristics of the patients are summarized in Supplementary Table S1.

### Cell lines and cell culture

Human colorectal cancer cell lines HCT116 cells (RRID: CVCL_0291; Catalog No. CL-0096) and SW480 cells (RRID: CVCL_0546; Catalog No. CL-0223) were purchased from Procell Life Science & Technology (Wuhan, China). Cell lines were authenticated by short tandem repeat (STR) profiling, and all lines tested negative for mycoplasma contamination. HCT116 cells were cultured in McCoy’s 5A medium (Procell) supplemented with 10% fetal bovine serum (FBS; Procell) and 1% penicillin-streptomycin solution (MedChemExpress, New Jersey, USA). SW480 cells were cultured in high-glucose Dulbecco’s modified Eagle’s medium (DMEM; Gibco, California, USA) containing 10% FBS and 1% penicillin-streptomycin solution. All cells were incubated at 37 °C in a humidified incubator with 5% CO_2_.

### Reagents and antibodies

RSL3 (Catalog No. B6095), CHIR-99021 (Catalog No. A3011), XAV-939 (Catalog No. A1877), Ferrostatin-1 (Fer-1; Catalog No. A4371), and D-luciferin potassium salt (Catalog No. C3654) were purchased from APExBIO (Houston, Texas, USA). Drug concentrations were determined based on the manufacturer’s instructions, previous studies, and preliminary experiments. Specifically, RSL3 was used at 2 μM for in vitro assays and 5 mg/kg for in vivo studies [21–23]; CHIR-99021 at 10 μM for in vitro experiments and 10 mg/kg for in vivo studies [24]; XAV-939 at 5 μM for in vitro experiments and 10 mg/kg for in vivo studies [25–27]; Fer-1 at 10 μM for in vitro experiments [28]; D-luciferin potassium salt was administered at 15 mg/kg for in vivo imaging. The FABP2 antibody (Catalog No. GTX83361) was obtained from GeneTex (San Antonio, Texas, USA), while the FABP2 antibody for co-immunoprecipitation (Co-IP) was purchased from Abways (Catalog No. BY2571, Shanghai, China). Antibodies against GPX4 (Catalog No. 67763-1-Ig) and β-catenin (Catalog No. 66379-1-Ig) were purchased from Proteintech (Wuhan, China). Detailed antibody dilutions used are provided in Supplementary Table S2.

### Cell proliferation assay

Cell proliferation was assessed using BeyoClick^TM^ EdU-594 Cell Proliferation Kit (Catalog No. C0078; Beyotime, Shanghai, China) according to the manufacturer’s instructions. Briefly, cells were seeded into confocal dishes and incubated with 10 μM EdU for 30 min, either after attachment or following 48 h of drug treatment. Subsequently, cells were fixed with 4% paraformaldehyde for 30 min and permeabilized with 0.3% Triton X-100 for 10 min. Incorporated EdU was visualized using the kit’s click reaction reagents by incubating in the dark at room temperature for 30 min. Nuclei were counterstained with DAPI-containing mounting medium (Servicebio, Wuhan, China) for 10 min. Fluorescence images were captured using a LSM710 laser confocal microscope (Carl Zeiss, Jena, Germany).

### Cell migration and invasion assay

Cell migration and invasion were assessed using Corning Transwell chambers (12-well format). For the invasion assay, the inserts were pre-coated with Matrigel (NEST, Wuxi, China). Briefly, Matrigel was diluted 1:8 (final concentration: 0.4875 μg/μL) in ice-cold serum-free medium. Then, 60 μL of the diluted Matrigel was evenly applied to the upper surface of the membrane and incubated at 37 °C for 3 h to allow gelation. After polymerization, the remaining liquid was removed, and the coated chambers were rehydrated with 100 μL serum-free medium for 30 min at 37 °C. Cells were starved in serum-free medium for several hours prior to the experiment. Following trypsinization, cells were resuspended in serum-free medium and adjusted to a density of 2 × 10^5^ cells/mL. A volume of 100–150 μL of cell suspension (approximately 2 × 10^4^ cells) was seeded into each Matrigel-coated upper chamber. The lower chamber was filled with a complete medium supplemented with 20% FBS serving as a chemoattractant. After incubation for 48 h at 37 °C in a humidified atmosphere containing 5% CO_2_, non-invading cells on the upper surface of the membrane were gently wiped off with a cotton swab. Cells that migrated to the lower surface were fixed with 4% paraformaldehyde for 15 min and stained with 0.1% crystal violet for 15 min. After washing with phosphate-buffered saline (PBS), images were captured under a microscope. The number of cells in five randomly selected fields per well was counted for statistical analysis. For the migration assay, uncoated Transwell inserts were used following the same protocol described above.

### Wound healing assay

Wound healing assays were conducted in 6-well plates (NEST, Wuxi, China). To facilitate image acquisition, three parallel reference lines were marked on the bottom of each well. Cells were seeded at a density of 2.5 × 10^6^ cells per well and cultured until reaching confluence. A linear wound was then made perpendicular to the reference lines using a sterile 200 μL pipette tip. After washing with PBS to remove detached cells, the monolayer was treated with the indicated drugs. Images of the wound area were captured at 0, 24, and 48 h using a phase-contrast microscope. The wound width was measured using ImageJ software (NIH, Bethesda, MD, USA), and the wound closure rate was calculated to quantify cell migration.

### Lipid peroxidation assay

Lipid peroxidation was assessed using BODIPY^TM^ 581/591 C11 Lipid Peroxidation Assay Kit (Catalog No. S0043S; Beyotime, Shanghai, China) following the manufacturer’s instructions. Briefly, cells were seeded into glass-bottom confocal dishes (NEST, Wuxi, China) and treated as indicated. After treatment, cells were washed twice with PBS and incubated with 2 μM probe working solution at 37 °C for 20 min in the dark. Subsequently, cells were washed with PBS and immediately imaged using a laser confocal microscope (LSM710; Carl Zeiss, Jena, Germany). Fluorescence was excited at 488 nm, and emission signals were collected at 510–550 nm (oxidized form, green) and 570–620 nm (reduced form, red). The ratio of green-to-red fluorescence intensity was calculated to quantify lipid peroxidation levels. Background fluorescence was subtracted before calculating the ratio.

### Measurement of intracellular reactive oxygen species (ROS)

Cells were seeded into glass-bottom confocal dishes (NEST, China) at a density of 1 × 10^5^ cells per dish and allowed to attach overnight. Subsequently, cells were treated with the indicated drugs for 48 h. After treatment, cells were incubated with the DHE-based ROS Assay Kit (Catalog No. C1300-2; Applygen, Beijing, China) at 37 °C for 30 min in the dark. Cells were then washed three times with warm PBS to remove unbound probe. Fluorescence images were captured using a laser scanning confocal microscope (LSM710; Carl Zeiss, Jena, Germany) with excitation at 518 nm and emission collected at 605 nm. The mean fluorescence intensity was analyzed to quantify intracellular ROS levels.

### Lipid peroxidation (malondialdehyde, MDA) assay

Intracellular lipid peroxidation was assessed by measuring malondialdehyde (MDA) levels using an MDA assay kit (Catalog No. S0131; Beyotime, Shanghai, China) according to the manufacturer’s instructions. Briefly, cells were lysed in RIPA buffer supplemented with protease inhibitors on ice for 30 min, followed by centrifugation at 12,000 × *g* for 10 min at 4 °C. The supernatants (100 μL) were mixed with 200 μL thiobarbituric acid (TBA) working solution and incubated at 100 °C for 15 min to form MDA-TBA adducts. After cooling to room temperature and subsequent centrifugation, the absorbance of the supernatant was measured at 532 nm using a microplate reader. MDA concentrations were calculated based on a standard curve and normalized to the total protein content, which was determined using a BCA protein assay kit. Results are expressed as μg MDA per mL protein.

### Measurement of intracellular ferrous iron (Fe^2+^) levels

Intracellular Fe^2+^ levels were determined using a Cellular Red Fluorescence Ferrous Ion Assay Kit (RhoNox-6, Catalog No. S1070; Beyotime, Shanghai, China) according to the manufacturer’s instructions. Briefly, cells were seeded into glass-bottom confocal dishes (NEST, Wuxi, China) and treated with the indicated compounds for 48 h. Following treatment, cells were incubated with a working solution containing RhoNox-6 (1×) and Hoechst 33342 (1×) in serum-free and phenol red-free culture medium at 37 °C for 30 min in the dark. Subsequently, cells were immediately imaged using a laser confocal microscope (LSM710; Carl Zeiss, Jena, Germany). RhoNox-6 fluorescence (red; Ex/Em = 545/585 nm) was used to visualize Fe^2+^ distribution, and Hoechst 33342 (blue; Ex/Em = 405/468 nm) served as a nuclear counterstain for cellular localization.

### Mitochondrial membrane potential assay

Mitochondrial membrane potential (ΔΨm) was assessed using a Mito-Tracker Red CMXRos probe (Catalog No. C1049B; Beyotime, Shanghai, China). Briefly, FABP2- overexpression and knockdown cell lines were seeded into glass-bottom confocal dishes (NEST, Wuxi, China) at a density of 1 × 10^6^ cells per dish and cultured until adherent. The working solution of Mito-Tracker Red CMXRos was freshly prepared by diluting a 200 μM stock solution (in DMSO) into pre-warmed complete culture medium to a final concentration of 100 nM. Cells were incubated with the staining solution at 37 °C in a humidified atmosphere containing 5% CO_2_ for 30 min. After incubation, the staining solution was removed, and cells were washed three times with pre-warmed PBS to remove unbound probe. Nuclei were counterstained with Hoechst 33342 (Beyotime, Shanghai, China) according to the manufacturer’s instructions. Fluorescence images were acquired immediately using a laser scanning confocal microscope (LSM710; Carl Zeiss, Jena, Germany). Mito-Tracker Red CMXRos was excited at 579 nm with emission collected at 599 nm, while Hoechst 33342 was excited at 405 nm with emission collected at 468 nm.

### Transmission electron microscopy (TEM)

To evaluate the effect of FABP2 expression on ferroptosis‑related mitochondrial morphology in colon cancer cells, transmission electron microscopy (TEM) was performed. Cells from all experimental groups (sh‑NC, sh‑FABP2, sh‑FABP2 + RSL3, oe‑NC, oe‑FABP2, oe‑FABP2 + Fer‑1) were fixed with 2.5% glutaraldehyde, followed by gradient ethanol dehydration, epoxy resin embedding, and polymerization. Ultrathin sections (∼70 nm) were prepared and stained with uranyl acetate and lead citrate (EMCN, Beijing, China). Observations were carried out using a JEM-1400FLASH transmission electron microscope (JEOL, Tokyo, Japan). Images were initially acquired at low magnification (10,000×) for cell localization, followed by systematic examination at intermediate to medium‑high magnification (25,000×). Key ferroptosis‑associated ultrastructural features, including mitochondrial cristae loss, mitochondrial shrinkage, increased membrane density, and outer membrane integrity alterations, were analyzed. At least 10 intact mitochondrial profiles per group were randomly selected from multiple fields and analyzed in a blinded manner to elucidate the ultrastructural basis of the effects of FABP2 knockdown (KD) and overexpression (OE) on ferroptosis sensitivity and protection.

### Immunohistochemistry (IHC) and quantification

Tissue specimens were fixed in 4% paraformaldehyde, embedded in paraffin, and sectioned at a thickness of 4 μM. Following deparaffinization and rehydration, antigen retrieval was performed by heating sections in citrate buffer (pH 6.0) in a microwave oven until boiling, followed by maintenance at a sub-boiling temperature for 10 min. Sections were then blocked with 5% bovine serum albumin (BSA) for 30 min at room temperature. Subsequently, slides were incubated overnight at 4 °C with primary antibodies, followed by incubation with HRP-conjugated secondary antibodies for 60 min at room temperature. Immunoreactivity was visualized using a DAB substrate kit (Catalog No. G1212; Servicebio, Wuhan, China), and sections were counterstained with hematoxylin. Images were acquired using an E100 bright-field microscope (Nikon, Tokyo, Japan). Quantification was performed by two independent observers in a blinded manner. The integrated optical density (IOD) of positively stained areas was measured in at least three randomly selected fields per section using ImageJ software (NIH, Bethesda, MD, USA). Immunohistochemistry scoring was performed electronically using ImageJ software (NIH, Bethesda, MD, USA) and the IHC Profiler plugin. At least three high-power fields (magnification) were blindly selected from whole-slide images for each section. After color deconvolution, the pixel intensity of DAB staining was automatically determined, and high-positive, moderate-positive, low-positive, and negative zones were calculated. The H-score was calculated as: H-score = (% high-positive × 3) + (% moderate-positive × 2) + (% low-positive × 1). The median H-score of the selected fields represented the final value for each section.

### Cell transfection

Lentiviral vectors for *FABP2* overexpression and knockdown were purchased from GeneChem (Shanghai, China). Cells were transduced with the corresponding lentiviruses in the presence of polybrene according to the manufacturer’s instructions. Stable cell lines were established by selecting transduced cells with puromycin (2 μg/mL). Transfection efficiency was validated by qRT-PCR or western blotting analysis. To ensure biological relevance and efficient gene modulation, knockdown experiments were conducted in cell lines with high endogenous *FABP2* expression, whereas overexpression experiments were performed in those with low endogenous levels.

### Chromatin immunoprecipitation (ChIP) assay

ChIP assays were performed using a commercial kit (Catalog No. 17-295; Millipore, Darmstadt, Germany) according to the manufacturer’s protocol with minor modifications. Briefly, cells were cross-linked with 1% formaldehyde for 20 min, quenched with 0.125 M glycine. After washing twice with ice-cold PBS, nuclei were isolated and lysed. Chromatin was fragmented into fragments of approximately 200-500 bp by sonication. Lysates were incubated overnight at 4 °C with specific antibodies against TCF4 (Catalog No. CY8810; Abways, China), histone H3 (Catalog No. ab1791; Abcam, USA), or control IgG (Catalog No. ab172730; Abcam, USA). Immune complexes were precipitated using Protein A magnetic beads (Catalog No. 16-661; Millipore). Following sequential washes with low-salt, high-salt, LiCl, and TE washes, protein-DNA complexes were eluted and reverse cross-linked. Purified DNA was analyzed by quantitative PCR (qPCR) using SYBR Premix Ex Taq (Catalog No. RR820A; Takara, USA) on a StepOne Plus Real-Time PCR System (Applied Biosystems, Massachusetts, USA). Enrichment of the target *TCF4* binding regions was calculated as a ratio relative to the input DNA.

### Dual-luciferase reporter assay

Dual-luciferase reporter assays were performed using a Dual-Luciferase Reporter Assay System (Catalog No. E1910; Promega, Madison, WI, USA) according to the manufacturer’s instructions. Briefly, sh-NC and sh-*FABP2* cells were cultured in DMEM supplemented with 10% fetal bovine serum and antibiotics (100 U/mL penicillin, 100 μg/mL streptomycin) at 37 °C in a humidified atmosphere containing 5% CO_2_. Cells were seeded into 24-well plates (5 × 10^4^ cells/well) and co-transfected with either the pGL3-*GPX4* plasmid or the empty pGL3-Basic control plasmid, together with 40 ng of the pRL-TK plasmid (Catalog No. D2760; Beyotime, Shanghai, China), using Lipofectamine 3000 reagent (Catalog No. L30000015; Invitrogen, Carlsbad, California, USA) following the manufacturer’s protocol. After 6 h, the culture medium was replaced with fresh complete medium, and cells were incubated for an additional 42 h. Cells were lysed using passive lysis buffer, and firefly and renilla luciferase activities were measured using a SpectraMax M5e microplate luminometer (Molecular Devices, San Jose, CA, USA). Firefly luciferase activity was normalized to renilla luciferase activity to correct for transfection efficiency. All experiments were performed in triplicate and repeated at least three independent times. Details regarding the construction and verification of the pGL3-*GPX4* promoter plasmid are provided in Supplementary File 1.

### Western blotting (WB) analysis

Cells and tissue samples were lysed in RIPA buffer (Catalog No. R0010; Solarbio, Beijing, China) supplemented with protease and phosphatase inhibitor cocktails (Catalog No. K1007 and K1012; APExBIO, Houston, TX, USA). Protein concentrations were determined using a BCA protein assay kit (Catalog No. PC0020; Solarbio, Beijing, China). Equal amounts of protein (20–40 μg) were separated by 10% or 12% SDS-PAGE (depending on the target molecular weight) and transferred onto PVDF membranes (Millipore, Billerica, MA, USA). Membranes were blocked with 5% non-fat milk in TBST (Tris-buffered saline with 0.1% Tween-20) for 1 h at room temperature, followed by overnight incubation at 4 °C with the specific primary antibodies (details listed in Supplementary Table S2). After washing with TBST, membranes were incubated with horseradish peroxidase (HRP)-conjugated secondary antibodies (Cell Signaling Technology, Danvers, MA, USA) for 1 h at room temperature. Protein bands were visualized using an Enhanced Chemiluminescence (ECL) Detection Kit (NCM Biotech, Suzhou, China) and captured using a ChemiScope 6200 Chemiluminescence and Fluorescence Imaging System (CLINX, Shanghai, China). Band intensities were quantified using ImageJ software (NIH, Bethesda, MD, USA). Full-length, uncropped blots for all experiments are provided in Supplementary Material-Original Blots.

### Co-immunoprecipitation (Co-IP)

Reciprocal co-immunoprecipitation assays were performed using a Commercial Co-IP Kit with Protein A/G magnetic beads (Catalog No. HY-K0202; MedChemExpress, New Jersey, USA) according to the manufacturer’s instructions. Briefly, cells were lysed in ice-cold IP buffer supplemented with protease and phosphatase inhibitor cocktails. The clarified lysates were incubated with the indicated primary antibodies or normal IgG (negative control) overnight at 4 °C with gentle rotation. Subsequently, Protein A/G magnetic beads were added to the lysates and incubated for an additional 4 h at 4 °C to capture the immune complexes. The beads were washed extensively (3–5 times) with ice-cold IP buffer to remove non-specific binding. Immune complexes were eluted by boiling in 2× SDS loading buffer for 10 min. The precipitated proteins, along with the input controls, were analyzed by SDS-PAGE, followed by Western blotting.

### Protein-protein docking and interaction analysis

The three-dimensional structures of FABP2 and β-catenin (encoded by CTNNB1) were retrieved from the Protein Data Bank (PDB) (Accession IDs: FABP2, 3AKM; β-catenin, 1G3J). Prior to docking, structures were prepared by removing water molecules, adding hydrogen atoms, and assigning charges using AutoDockTools developed by the Olson laboratory [29]. Protein-protein docking was performed using the local version of HDOCK software (version 1.1; Huang Lab, Huazhong University of Science and Technology, Wuhan, China), which employs a hybrid algorithm combining physical and bioinformatics approaches to sample possible binding modes and rank them via a scoring function. The top-ranked docking poses were further evaluated by calculating the binding free energy using the MM/GBSA method implemented in the HawkDock server (http://cadd.zju.edu.cn/hawkdock/). To characterize non-covalent interactions at the binding interface, the Protein-Ligand Interaction Profiler (PLIP) web server was utilized. Interactions including hydrogen bonds, salt bridges, and hydrophobic contacts were identified based on standard geometric criteria: hydrogen bond distance ≤4.1 Å and donor-acceptor-hydrogen angle ≥100°; salt bridge distance ≤5.5 Å between oppositely charged centers. All molecular graphics and interaction details were visualized and confirmed using PyMOL software (version 2.5; Schrödinger, LLC, New York, NY, USA).

### Surface plasmon resonance (SPR) analysis

SPR experiments were performed using a Biacore 1K instrument (Cytiva, Uppsala, Sweden) at 25 °C. Recombinant human FABP2 was immobilized on a CM5 sensor chip (Cytiva) via standard amine coupling. 1.0× PBS‑P⁺ (pH 7.4) was used as the running buffer. Briefly, the active flow cell (Channel 2) was activated with a 1:1 (v/v) mixture of 0.4 M EDC and 0.1 M NHS at a flow rate of 10 μL/min for 420 s, achieving an immobilization level of approximately 8143.2 response units (RU). Remaining active esters were deactivated with 1 M ethanolamine‑HCl (pH 8.5) at 10 μL/min for 420 s. A reference flow cell (Channel 1) was prepared identically but without protein injection (mock coupling). Kinetic analyses were performed using serial two-fold dilutions of recombinant human β-catenin (encoded by *CTNNB1*) ranging from 0.125 to 128 μM in the running buffer (1× PBS-P⁺ (pH 7.4) supplemented with 5% (v/v) DMSO). Analytes were injected over both flow cells at a flow rate of 30 μL/min for an association time of 100 s, followed by a dissociation phase of 120 s. The sensor chip surface was regenerated between cycles by injecting 10 mM glycine-HCl (pH 2.0) for 5 min. Sensorgrams were reference-subtracted and globally fitted to a 1:1 Langmuir binding model using the Biacore 1K Evaluation Software to determine the association rate constant (*k*_*a*_), dissociation rate constant (*k*_*d*_), and equilibrium dissociation constant (*K*_*D*_=*k*_*d*_*/k*_*a*_).

### Immunoprecipitation-mass spectrometry (IP-MS)

For immunoprecipitation, SW480 cells stably overexpressing FABP2 were harvested and lysed in ice-cold IP lysis buffer supplemented with protease and phosphatase inhibitors. Clarified lysates containing 2 mg of total protein were incubated overnight at 4 °C with 4 μg of anti-β-catenin antibody (Catalog No. 66379-1-Ig; Proteintech, Wuhan, China) under gentle rotation. Subsequently, 50 μL of Protein A/G magnetic beads (Catalog No. HY-K0202; MedChemExpress, New Jersey, USA) were added and incubated for 4 h at 4 °C to capture the immune complexes. After extensive washing with lysis buffer, bead-bound proteins were eluted using 2× SDS loading buffer by boiling for 10 min. The immunoprecipitated samples were subjected to liquid chromatography-tandem mass spectrometry (LC-MS/MS) analysis at Shanghai Applied Protein Technology Co., Ltd. (Shanghai, China). Proteins were identified against the UniProt human database using MaxQuant software (version 1.6.14; ‌Max Planck Institute of Biochemistry, Martinsried, Germany). Trypsin was used for digestion, and carbamidomethylation of cysteine and oxidation of methionine were set as fixed and variable modifications, respectively. Peptide and protein identifications were filtered at a false discovery rate (FDR) of <1%.

### RNA sequencing and data analysis

Total RNA was extracted from colon cancer tissues and matched adjacent normal tissues using TRIzol reagent (Invitrogen, Carlsbad, CA, USA). RNA concentration and purity were assessed using a NanoDrop 2000 spectrophotometer, and RNA integrity was evaluated using an Agilent 2100 Bioanalyzer. Samples with an RNA integrity number (RIN) ≥ 7.0 were selected for sequencing. The mRNA was purified using oligo (dT) magnetic beads, fragmented, and reverse-transcribed into cDNA. Following end repair, adapter ligation, and PCR enrichment, the libraries were validated and sequenced on an Illumina NovaSeq 6000 platform (paired-end, 150 bp reads) by YASEN Biotechnology (Wuhan, China). Raw reads were quality-filtered using Cutadapt and aligned to the human reference genome (GRCh38) using HISAT2. Gene counts were obtained with HTSeq, and differential expression analysis was performed using DESeq2, with |*log*_*2*_*(fold change)*| > 1 and an adjusted *p-value* < 0.05 set as significance thresholds. Functional enrichment analyses, including Gene Ontology (GO), Kyoto Encyclopedia of Genes and Genomes (KEGG), and Gene Set Enrichment Analysis (GSEA), were conducted using the clusterProfiler R package. Protein-protein interaction (PPI) networks were constructed using the STRING database (https://string-db.org/) [30] and visualized with Cytoscape software (version 3.10.3; Cytoscape Consortium, Boston, USA).

### Immunofluorescence (IF) and colocalization analysis

Cells were seeded into confocal dishes at a density of 2.5 × 10^5^ cells per well and subjected to immunofluorescence staining. Cells were fixed with 4% paraformaldehyde for 10 min at room temperature, permeabilized with 0.1% Triton X-100 in PBS for 10 min, and blocked with 5% BSA for 1 h to prevent non-specific binding. Subsequently, cells were incubated overnight at 4 °C with primary antibodies derived from different host species. After washing with PBS, cells were incubated with species-specific secondary antibodies conjugated to AlexaFluor594 for 1 h at room temperature in the dark. Nuclei were counterstained with DAPI. Fluorescence images were acquired using a laser scanning confocal microscope (LSM710; Carl Zeiss, Jena, Germany) equipped with a 63× objective. Colocalization analysis was performed using ImageJ software (NIH, Bethesda, MD, USA) to calculate Pearson’s correlation coefficients between the fluorescence channels.

### Animal studies

All animal experiments were conducted in accordance with institutional ethical guidelines and were approved by the Animal Experiment Ethics Committee of Guizhou Medical University (Approval Number: 2502953). Male BALB/c nude mice (5–6 weeks old, weighing 18–20 g) were purchased from Chengdu GemPharmatech Biotechnology Co., Ltd. (Chengdu, China) and maintained under specific pathogen-free (SPF) conditions in the animal facility of the Clinical Research Center, Affiliated Hospital of Guizhou Medical University, with six mice per cage. All animals were randomized using a random number table before surgery, and the procedures were performed by the same experienced investigator using standardized surgical protocols. Mice were weighed and anesthetized with Tribromoethanol (20 μL/g; Catalog No. PMK2009; Bioprimacy, Hubei, China). FABP2-knockdown HCT116 cells (5 × 10^6^ in 100 μL PBS) carrying a luciferase tag, along with control cells, were injected into mice via the portal vein (40 mice in the experimental group and six in the control group). Following portal vein injection, mortality was observed in 15 mice from the experimental group and one mouse from the control group. All deaths occurred within the early postoperative period, potentially attributable to anesthesia-related complications or perioperative hypothermia. In vivo bioluminescence imaging was performed on day 7 after injection to confirm successful establishment of the liver metastasis model. Mice with confirmed liver tumor formation (*n* = 20) were randomly divided into five treatment subgroups (*n* = 4 per group) and received daily intraperitoneal injections of XAV-939 (10 mg/kg), RSL3 (5 mg/kg), CHIR-99021 (10 mg/kg), RSL3 (5 mg/kg) + CHIR-99021 (10 mg/kg) (administered separately via intraperitoneal injection), or vehicle control for 14 consecutive days. Imaging was repeated on days 7 and 14 of treatment to monitor tumor progression. Body weights and clinical signs were monitored daily during the first week post-surgery and twice weekly thereafter. On day 22 after portal vein injection, all mice were euthanized, and liver tissues were collected for histological and molecular analyses. The experimental timeline is shown in Supplementary Fig. S3H. We used the ARRIVE reporting guideline [31] to draft this manuscript, and the ARRIVE reporting checklist during editing, which is included in the Supplementary File 2: ARRIVE-checklist.

### In vivo bioluminescence imaging in mice

Mice were anesthetized with 2–3% isoflurane (Catalog No. R510-22; RWD, Shenzhen, China) in oxygen, and body weights were recorded prior to imaging. D-luciferin potassium salt (Catalog No. C3654; APExBIO, Houston, Texas, USA) was administered via intraperitoneal injection at a dose of 150 mg/kg. Mice were placed in a supine position on the heated stage of an IVIS LUMINA III in vivo imaging system (PerkinElmer, Waltham, MA, USA) and maintained at 37 °C to prevent hypothermia. Bioluminescent images were acquired 10–15 min after substrate administration, corresponding to the peak signal intensity for intraperitoneal injection. Regions of interest (ROIs) were manually drawn around the liver area for each mouse using Living Image 4.4 software (PerkinElmer, Waltham, MA, USA). Total photon flux (photons/sec) was quantified within each ROI after subtracting background bioluminescence from a region with no signal. All imaging analyses were performed by an investigator blinded to the treatment groups.

### Statistical analysis

All statistical analyses were performed using GraphPad Prism software (version 9.5.1; GraphPad Software, La Jolla, CA, USA). Data are representative of at least three independent biological experiments and are presented as mean ± standard deviation (SD), unless otherwise stated. Normality of data distribution was assessed using the *Shapiro–Wilk* test, and homogeneity of variances was evaluated by *Levene’s* test. For comparisons between two groups, the unpaired two-tailed *Student’s t-test* was used for normally distributed data, while the *Mann–Whitney U* test was applied for non-normally distributed data. For comparisons among three or more groups, *one-way analysis of variance* (*ANOVA*) followed by *Tukey’s* post hoc test was employed for data with normal distribution and equal variances. In cases where data did not meet the assumptions of normality or homogeneity, the *Kruskal–Wallis* test followed by *Dunn’s* post hoc test was used. All tests were two-tailed, and a *P-value* < 0.05 was considered statistically significant. Specific statistical details for each experiment, including the exact *P*-values and sample sizes (*n*), are provided in the corresponding figure legends.

## Results

### FABP2 downregulation is associated with tumor progression and distant metastasis in colon cancer

**Fig. 1:**
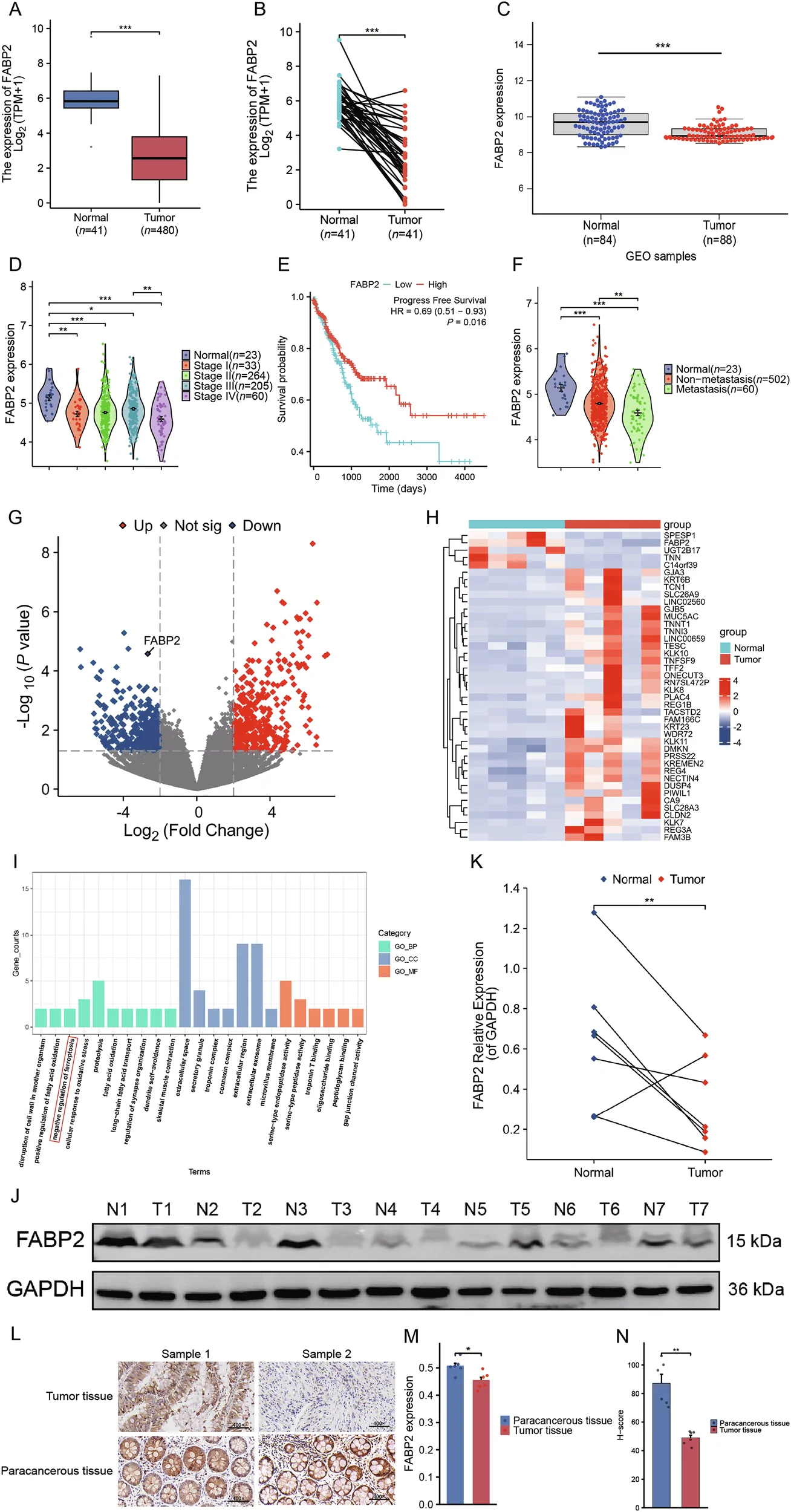
FABP2 expression is downregulated in colon cancer and associated with tumor progression, distant metastasis, and negative regulation of ferroptosis. **A**–**D** Bioinformatic analysis of FABP2 expression in colon cancer tissues reveals a marked downregulation in primary tumors compared with normal tissues, with expression levels inversely correlated with tumor stage. **E** Kaplan–Meier survival analysis shows that low FABP2 expression is associated with significantly poorer progression-free survival (PFS) in patients with colon cancer. **F** FABP2 expression is significantly lower in primary tumors from patients who develop distant metastases than in those without metastasis. **G**, **H** Transcriptomic profiling identifies FABP2 as one of the most significantly downregulated genes in colon cancer specimens. **I** Gene set enrichment analysis (GSEA) of differentially expressed genes reveals significant enrichment in pathways related to the negative regulation of ferroptosis. **J**, **K** Western blot analysis confirms markedly reduced FABP2 protein levels in tumor tissues compared with adjacent normal tissues (*n* = 7). **L**–**N** Immunohistochemical staining shows predominant cytoplasmic localization of FABP2 and confirms its significantly decreased expression in colon cancer tissues(*n* = 6). Statistical analyses: **A**–**C**, **K**, **M**, **N** Two-tailed *t*-test, data presented as mean ± SD; **D**, **F** Kruskal–Wallis test with Dunn’s post hoc test, data presented as median with interquartile range (IQR); **E** Cox proportional hazards model; Significance levels: *P < 0.05; **P < 0.01; ***P < 0.001.

To identify genes potentially involved in colon cancer progression, we performed bioinformatics analyses and observed reduced FABP2 expression in primary colon cancer tissues, with expression levels associated with tumor stage (Fig. 1A–D). Survival analysis further showed that lower FABP2 expression was associated with poorer progression-free survival (PFS) (Fig. 1E). To further explore the potential relationship between FABP2 and metastatic progression, analysis of a GEO dataset showed that patients who developed distant metastases during follow-up exhibited lower FABP2 expression in primary tumors than patients without metastasis (Fig. 1F). Consistent with these findings, transcriptome analysis of human colon cancer specimens identified FABP2 among the most markedly downregulated genes (Fig. 1G, H). Functional enrichment analysis of differentially expressed genes revealed enrichment of pathways related to ferroptosis regulation (Fig. 1I). Western blot analysis further confirmed reduced FABP2 protein expression in primary colon cancer tissues (Fig. 1J, K). Immunohistochemical staining showed predominantly cytoplasmic localization of FABP2, with weaker staining intensity in tumor tissues than in adjacent normal tissues (Fig. 1L–N).

Collectively, these findings suggest that FABP2 downregulation is associated with tumor progression and distant metastasis in colon cancer, and support a potential role for FABP2 in metastatic progression.

### FABP2 suppresses liver metastasis of colon cancer by enhancing ferroptosis sensitivity

Bioinformatics enrichment analysis suggested a potential association between FABP2 and ferroptosis-related pathways in colon cancer. To further investigate the role of FABP2 in ferroptosis regulation, a series of in vitro and in vivo experiments was performed.

**Fig. 2:**
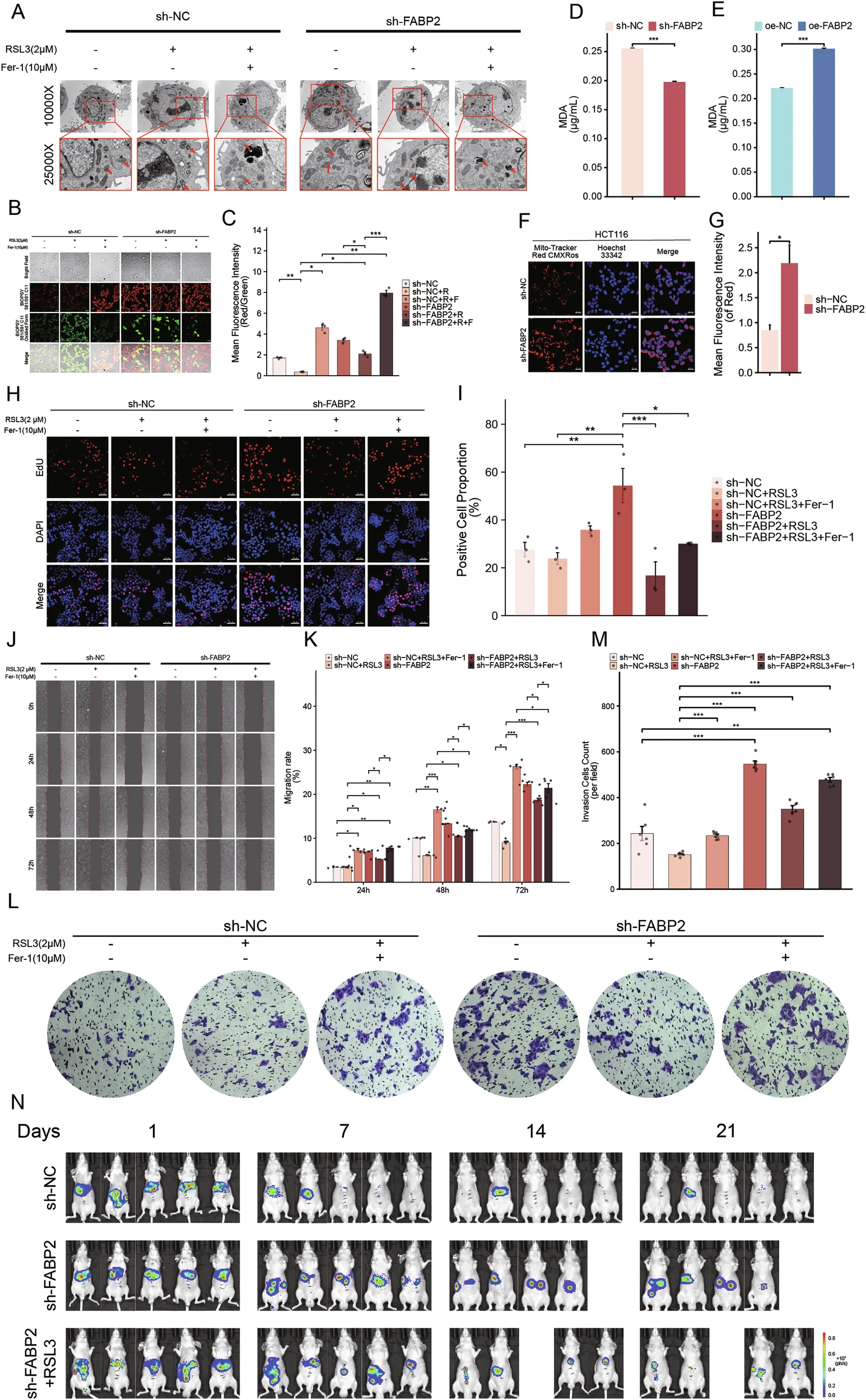
FABP2 suppresses colon cancer liver metastasis by enhancing ferroptosis sensitivity. **A** Transmission electron microscopy (TEM) images of HCT116 cells infected with sh-NC or sh-FABP2, treated with or without RSL3 (2 μM) and/or Fer-1 (10 μM). Red arrows indicate mitochondria displaying characteristic morphological features of ferroptosis. Knockdown of FABP2 attenuated RSL3-induced mitochondrial shrinkage and membrane density loss, indicating enhanced ferroptosis evasion. **B**, **C** Quantification of intracellular lipid peroxidation (LPO) levels in HCT116 cells with FABP2 knockdown or overexpression, with or without RSL3/Fer-1 treatment(*n* = 3). **D**, **E** Quantification of intracellular malondialdehyde (MDA) content in HCT116 cells with FABP2 knockdown (**D**) or SW480 cells with FABP2 overexpression (**E**) (*n* = 5). **F**, **G** Quantification of mitochondrial membrane potential assessed by Mito-Tracker Red CMXRos fluorescence in HCT116 cells with FABP2 knockdown(*n* = 3). **H**–**M** Functional assays in HCT116 cells with FABP2 knockdown, with or without RSL3/Fer-1 treatment(*n* = 3). EdU proliferation assays (**H**, **I**), wound healing migration assays (**J**, **K**), and Transwell invasion assays (**L**, **M**). Knockdown of FABP2 significantly enhanced proliferation, migration, and invasion, effects that were reversed by RSL3 treatment. Subsequent addition of Fer-1 restored the malignant phenotype in FABP2-knockdown cells treated with RSL3. **N** Representative bioluminescence images in mice injected with sh-NC or sh-FABP2 HCT116 cells, with or without RSL3 treatment (*n* = 5). FABP2 knockdown increased liver metastasis, which was suppressed by RSL3. Scale bars: **A** 1 μm; **B**, **F** 20 μm; **H** 50 μm. Statistical analyses: **D**, **E** two-tailed *t*-test, data presented as mean ± SD; **C**, **G**, **I**, **K**, **M** Kruskal–Wallis test with Dunn’s post hoc test, data presented as median with interquartile range (IQR); Significance levels: ns not significant; *P < 0.05, **P < 0.01, ***P < 0.001.

Transmission electron microscopy (TEM) revealed reduced ferroptosis-associated mitochondrial changes in FABP2-knockdown cells compared with control cells, including less prominent mitochondrial shrinkage, preserved cristae structure, and lower membrane density. Treatment with the ferroptosis inducer RSL3 partially restored these morphological features (Fig. 2A). In line with these observations, FABP2 knockdown reduced intracellular reactive oxygen species (ROS), lipid peroxidation, and malondialdehyde (MDA) levels and increased mitochondrial membrane potential, as indicated by Mito-Tracker fluorescence (Fig. 2B–G). Conversely, FABP2 overexpression enhanced ferroptosis-associated changes (Supplementary Fig. S1A, B, Supplementary Fig. S3A, B). Intracellular Fe^2+^ accumulation was detected across experimental groups, although no significant differences were observed (Supplementary Fig. S1C, D). Therefore, we hypothesize that the mechanism by which FABP2 induces ferroptosis likely relies on lipid metabolic pathways or modulation of the cellular antioxidant system, rather than an increase in the labile iron pool.

Functionally, FABP2 knockdown enhanced the proliferative, migratory, and invasive capacities of colon cancer cells, whereas FABP2 overexpression suppressed these malignant phenotypes (Fig. 2H–M, Supplementary Fig. S1C–H). Induction of ferroptosis with RSL3 partially reversed the enhanced proliferation, migration, and invasion induced by FABP2 knockdown, whereas subsequent treatment with the ferroptosis inhibitor ferrostatin-1 (Fer-1) restored these malignant phenotypes (Fig. 2H–M, Supplementary Fig. S1C–H). These findings support a role for ferroptosis in mediating the biological effects of FABP2.

Given that liver metastasis represents a critical step in the distant spread of colon cancer [32], we further assessed the role of FABP2 in metastatic colonization in vivo. Mice injected with FABP2-knockdown colon cancer cells developed a greater liver metastatic burden, whereas RSL3 treatment attenuated this effect (Fig. 2N). Consistently, immunohistochemical staining showed increased Ki67 and PCNA in metastatic lesions following FABP2 knockdown, indicating enhanced proliferative activity (Supplementary Fig. S1I).

Together, these findings suggest that FABP2 suppresses colon cancer progression and liver metastasis, at least in part, through regulation of ferroptosis sensitivity.

### FABP2 enhances ferroptosis sensitivity by downregulating GPX4 expression

**Fig. 3:**
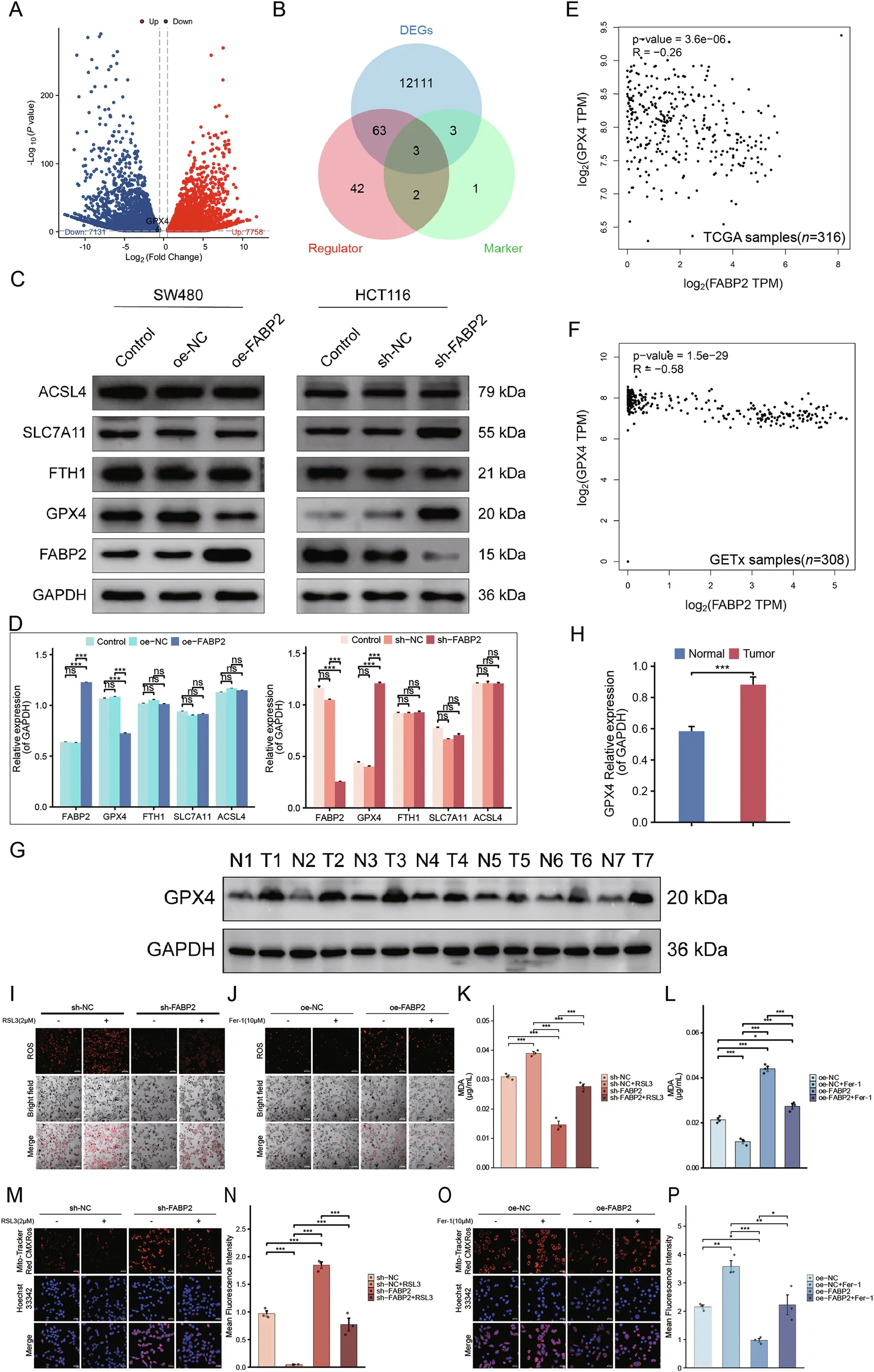
FABP2 enhances ferroptosis sensitivity by Downregulating GPX4 Expression. **A**, **B** Transcriptome sequencing of FABP2-overexpressing colon cancer cells identified differentially expressed genes (DEGs). Intersection analysis with known ferroptosis regulators from the FerrDb2 database revealed three overlapping genes, with GPX4 being a prominent negative regulator of ferroptosis. The volcano plot shows the distribution of DEGs (**A**); The Venn diagram shows the distribution of overlapping genes (**B**). **C**, **D** Western blot analysis of FABP2 knockdown (sh-FABP2) and overexpression (oe-FABP2) in SW480 and HCT116 cells demonstrated that GPX4 expression was significantly upregulated upon FABP2 depletion and downregulated upon FABP2 overexpression. Among the ferroptosis-related proteins tested (ACSL4, SLC7A11, FTH1, GPX4), GPX4 showed the most consistent response to FABP2 modulation. **E**, **F** Database analysis of human colon cancer tissues (TCGA, *n* = 316; GTEx, *n* = 308) showed a significant negative correlation between FABP2 and GPX4 expression levels, supporting this regulatory relationship. **G**, **H** Western blot analysis of paired normal and tumor tissues from colon cancer patients confirmed elevated GPX4 protein levels in tumor tissues compared to adjacent normal tissues(*n* = 7) (**G**). The bar graph shows quantification of GPX4 expression normalized to GAPDH (**H**). **I**–**P** Functional validation using the GPX4 regulator RSL3 (2 μM) or Fer-1 (10 μM) in FABP2-knockdown and overexpression cells. RSL3 treatment induced ferroptosis in FABP2-knockdown cells, as evidenced by increased ROS levels, malondialdehyde (MDA) levels, and enhanced Mito-Tracker fluorescence, indicating mitochondrial activity changes (*n* = 3). Scale bars: **I**, **J** 100 μm; **M**, **O** 20 μm. Statistical analyses: **H** two-tailed *t*-test, data shown as mean ± SD; **D**, **K**, **L**, **N**, **P** Kruskal–Wallis test with Dunn’s post hoc test, data shown as median with interquartile range (IQR); Significance levels: ns not significant, *P < 0.05, **P < 0.01, ***P < 0.001.

To investigate the molecular mechanism by which FABP2 regulates ferroptosis, transcriptomic analysis was performed in colon cancer cells overexpressing FABP2 to identify differentially expressed genes. These genes were intersected with established ferroptosis regulators and marker genes from the Ferroptosis Database (FerrDb2) [33], resulting in three overlapping candidates. Among them, glutathione peroxidase 4 (GPX4), a well-characterized suppressor of ferroptosis, emerged as a candidate downstream target of FABP2 (Fig. 3A, B).

Further analyses in FABP2 knockdown and overexpression models showed that FABP2 knockdown increased GPX4 expression, whereas FABP2 overexpression reduced GPX4 expression. Among the ferroptosis-related proteins examined, GPX4 exhibited the most consistent change in response to FABP2 modulation (Fig. 3C, D). Consistent with these observations, analysis of human colon cancer datasets showed an inverse correlation between FABP2 and GPX4 expression (Fig. 3E, F). Western blot analysis further showed increased GPX4 protein expression in colon cancer tissues compared to adjacent normal tissues (Fig. 3G, H).

To assess the contribution of GPX4 inhibition to ferroptosis-related phenotypes, FABP2-knockdown cells were treated with the GPX4 inhibitor RSL3. RSL3 treatment increased intracellular ROS, LPO, and MDA levels and altered mitochondrial membrane potential, as assessed by Mito-Tracker fluorescence (Fig. 3I–P). No significant differences in intracellular Fe^2+^ levels were observed among experimental groups (Supplementary Fig. S3C, D).

Collectively, these findings suggest that FABP2 may enhance ferroptosis sensitivity in colon cancer by downregulating GPX4, which appears to act as a key downstream mediator of FABP2-mediated tumor suppression.

### FABP2 regulates GPX4 expression through the Wnt/β-catenin signaling

**Fig. 4:**
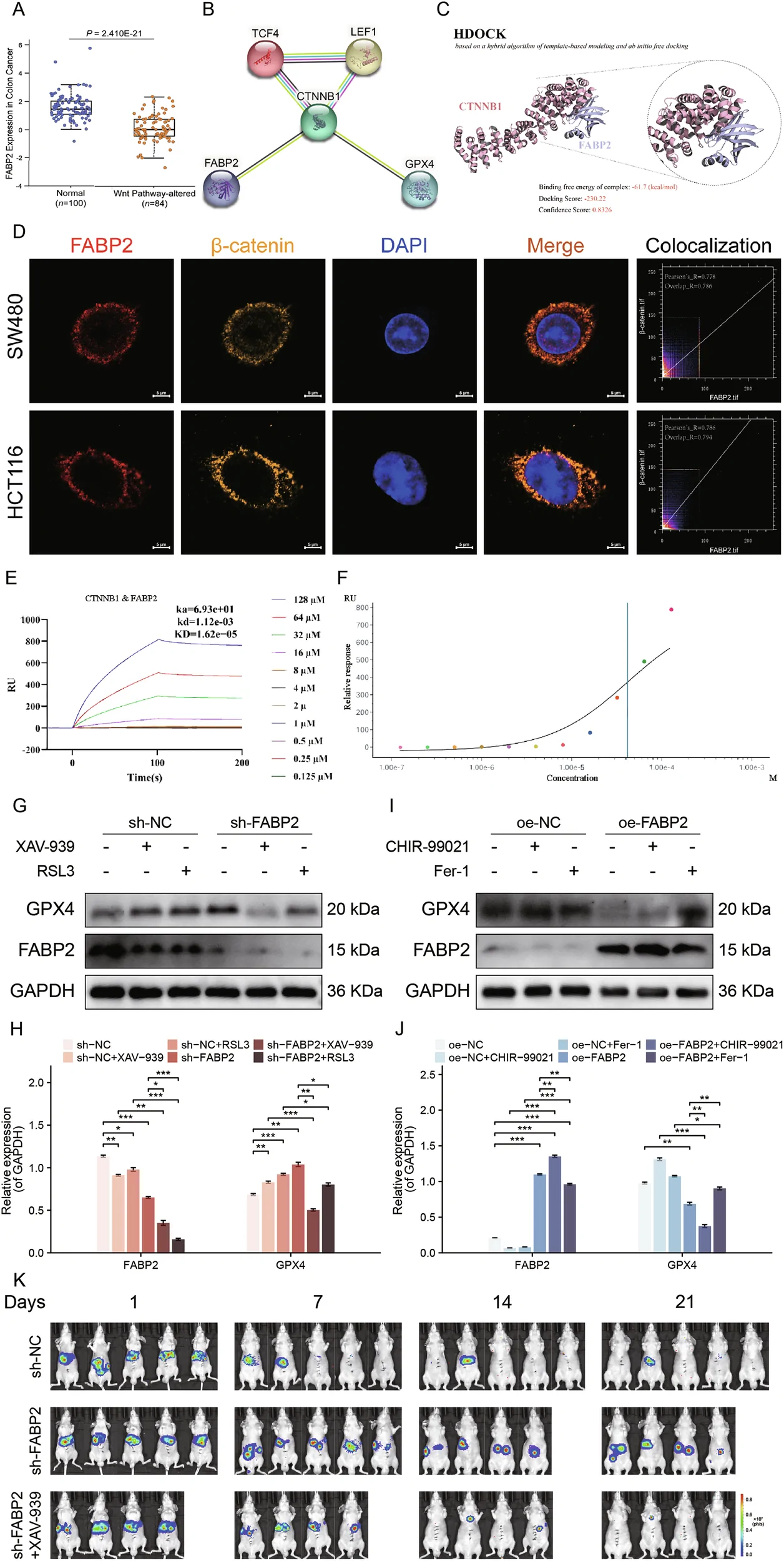
FABP2 regulates GPX4 expression through the Wnt/β-catenin signaling. **A** Bioinformatic analysis revealed an association between FABP2 expression and the Wnt signaling pathway in colon cancer. The STRING database (**B**) and HDOCK protein-protein docking analysis (**C**) predicted an interaction between FABP2 and CTNNB1 (β-catenin), showing a binding free energy of −61.7 kcal/mol and a docking score of −230.22. **D** Immunofluorescence colocalization assay confirmed the colocalization of FABP2 and β-catenin in the cytoplasm and nucleus of colon cancer cells. **E**, **F** Surface plasmon resonance (SPR) analysis validated the direct interaction between CTNNB1 and FABP2, with a calculated equilibrium dissociation constant (*K*_*D*_) of 1.62 × 10^-5 ^M. The representative sensorgrams are shown in (**F**). Western blot (**G**, **I**) and quantitative analyses (**H**, **J**, *n* = 3) demonstrated that knockdown of FABP2 increased GPX4 expression, which was partially reversed by treatment with the Wnt inhibitor XAV-939 (**G**, **H**). Conversely, overexpression of FABP2 reduced GPX4 levels, and this effect was counteracted by the Wnt activator CHIR-99021 or the ferroptosis inhibitor Fer-1 (**I**, **J**). **K** In vivo liver metastasis assay showed that XAV-939 treatment partially attenuated the enhanced liver metastatic potential of FABP2-knockdown colon cancer cells (*n* = 5). Scale bars**:** **D** 5 μm. Statistical analyses: **H**, **J** One-way ANOVA followed by Tukey’s post hoc test, data shown as median with interquartile range (IQR); Significance levels: ns, not significant; *P < 0.05; **P < 0.01; ***P < 0.001.

To investigate how FABP2 regulates GPX4 expression, we first examined whether FABP2 directly interacts with GPX4. Co-immunoprecipitation analysis did not detect an interaction between FABP2 and GPX4 (Supplementary Fig. S3E). Bioinformatic analysis indicated an association between FABP2 expression and Wnt signaling (Fig. 4A), whereas transcriptomic analysis identified *CTNNB1* (Encoding β-catenin protein) as a potential downstream mediator of FABP2 (Supplementary Fig. S2C, D). Protein interaction analysis using the STRING database (https://string-db.org/) [34] and protein-protein docking suggested a potential interaction between FABP2 and β-catenin (Fig. 4B, C, Supplementary Fig. S2A, B). This association was further supported by immunofluorescence colocalization, reciprocal co-immunoprecipitation, and surface plasmon resonance (SPR) analysis (Fig. 4D–F, Supplementary Fig. S2E).

Because nuclear β-catenin forms a transcriptional complex with TCF4 to regulate downstream target genes [35, 36], we next examined whether FABP2 influences GPX4 transcription through this pathway. ChIP-qPCR analysis showed increased TCF4 occupancy at the GPX4 promoter following FABP2 knockdown (Supplementary Fig. S2F). Consistently, dual-luciferase reporter assays showed increased GPX4 transcriptional activity after FABP2 downregulation (Supplementary Fig. S2G).

To further assess the involvement of Wnt signaling, FABP2-knockdown cells were treated with the Wnt pathway inhibitor XAV-939. XAV-939 partially attenuated GPX4 upregulation induced by FABP2 knockdown (Fig. 4G–J). Similarly, treatment with XAV-939 reduced the increased liver metastatic burden observed in mice injected with FABP2-knockdown cells (Fig. 4K, Supplementary Fig. S2H).

Overall, these results suggest that FABP2 regulates GPX4 expression, at least in part, through Wnt/β-catenin signaling and thereby contributes to ferroptosis-related regulation in colon cancer.

### FABP2 associates with β-catenin and restricts its nuclear translocation

**Fig. 5:**
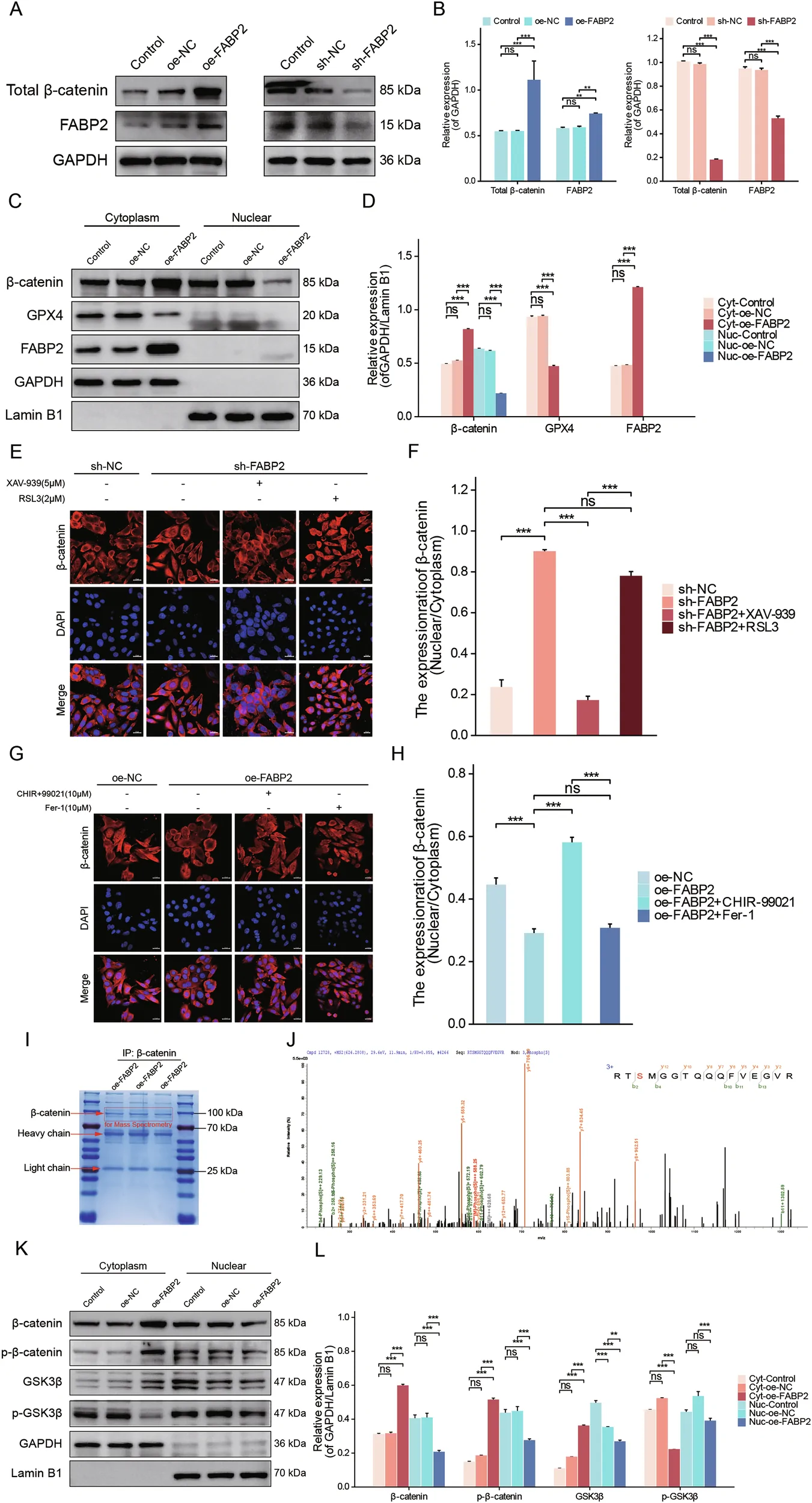
FABP2 associates with β-catenin and restricts its nuclear translocation. Western blot analysis (**A**) and quantification of total β-catenin levels (**B**) in control and FABP2-overexpressing (oe-FABP2) colon cancer cells. FABP2 overexpression significantly increased total β-catenin protein expression (*n* = 3). **C**, **D** Nuclear and cytoplasmic fractionation assays showing that FABP2 overexpression markedly reduced β-catenin levels in the nuclear fraction while increasing its cytoplasmic retention (*n* = 3). **E**–**H** Representative immunofluorescence images and corresponding quantification demonstrating that FABP2 overexpression substantially decreased the nuclear localization of β-catenin (*n* = 3). **I**, **J** Immunoprecipitation-mass spectrometry (IP-MS) analysis of Coomassie Brilliant Blue‑stained IP gel bands revealed that FABP2 overexpression enhanced β-catenin phosphorylation (Ser675). **K**, **L** Western blot analysis of phosphorylated β-catenin and phosphorylated GSK3β in nuclear and cytoplasmic fractions indicated that FABP2 promotes cytoplasmic β-catenin phosphorylation, with a non-synchronous change in GSK3β phosphorylation (*n* = 3). Scale bar: **E**, **G** 20 μm. Statistical analyses: Kruskal–Wallis test with Dunn’s post hoc test; data are presented as median with interquartile range (IQR); Significance levels: ns, not significant; *P < 0.05, **P < 0.01, ***P < 0.001.

To examine the effect of FABP2 on β-catenin, total β-catenin protein levels were assessed in FABP2 overexpression (FABP2-OE) colon cancer cells by western blotting. FABP2 overexpression increased total β-catenin protein levels (Fig. 5A, B).

Because β-catenin exerts transcriptional activity primarily through nuclear accumulation [37, 38], we next examined whether FABP2 affects its subcellular localization. Nuclear-cytoplasmic fractionation analysis showed reduced nuclear β-catenin levels in FABP2-OE cells, accompanied by increased cytoplasmic retention (Fig. 5C, D). Consistent with these findings, immunofluorescence staining demonstrated decreased nuclear localization of β-catenin following FABP2 overexpression (Fig. 5E–H). These results indicate that FABP2 alters the intracellular distribution of β-catenin despite increasing total protein abundance.

To explore the mechanism underlying this, immunoprecipitation-mass spectrometry (IP-MS) was performed using protein bands isolated from Coomassie Brilliant Blue–stained gels following immunoprecipitation (Fig. 5I). IP-MS analysis identified increased phosphorylation of β-catenin at Ser675 in FABP2-OE cells (Fig. 5J). Nuclear-cytoplasmic fractionation further showed increased cytoplasmic levels of phosphorylated β-catenin following FABP2 overexpression. Because β-catenin phosphorylation is influenced by multiple upstream kinases, including GSK3β, we next examined GSK3β phosphorylation. FABP2 overexpression increased cytoplasmic GSK3β phosphorylation, whereas nuclear levels remained relatively unchanged (Fig. 5K, L, Supplementary Fig. S3F, G).

In summary, these findings suggest that FABP2 limits β-catenin nuclear accumulation and may influence β-catenin signaling through altered cytoplasmic retention and phosphorylation.

### FABP2 modulates ferroptosis-related phenotypes through the Wnt/β-catenin/GPX4 signaling axis

**Fig. 6:**
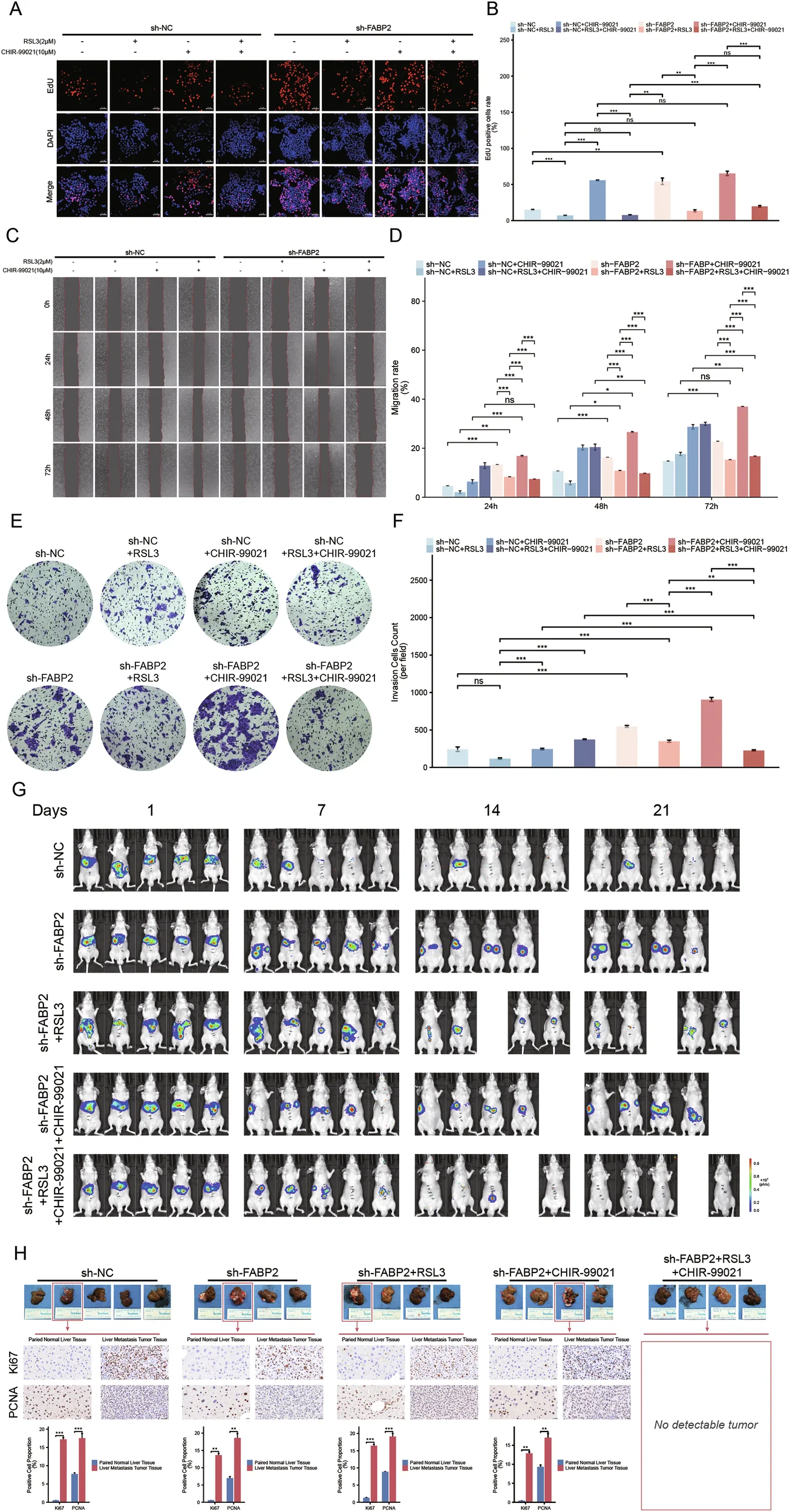
FABP2 suppresses ferroptosis evasion via the Wnt/β‑catenin/GPX4 signaling axis to inhibit colon cancer liver metastasis. **A**, **B** EdU proliferation assay in control (sh-NC), FABP2-knockdown (sh-FABP2) cells, and sh-FABP2 cells treated with the GPX4 inhibitor RSL3 (sh-FABP2+RSL3), the Wnt activator CHIR-99021 (sh-FABP2+CHIR-99021), or both (sh-FABP2+RSL3+CHIR-99021). Activation of the Wnt pathway failed to enhance proliferation in FABP2‑knockdown cells when GPX4 was inhibited by RSL3 (*n* = 6). **C**, **D** Wound healing assay performed under the same experimental conditions as in the EdU proliferation assay. CHIR‑99021 did not rescue migration in FABP2‑knockdown cells in the presence of RSL3 (*n* = 6). **E**, **F** Transwell invasion assay assessing invasive capacity of the indicated groups. Consistent with migration, Wnt activation did not promote invasion in FABP2‑knockdown cells treated with RSL3 (*n* = 6). **G**, **H** In vivo bioluminescence imaging of liver metastases in the five groups (*n* = 5). GPX4 inhibition by RSL3 partially reversed the pro‑metastatic effect induced by FABP2 knockdown, whereas Wnt activation failed to restore metastatic potential under GPX4 inhibition. No detectable tumor was observed in the sh-FABP2+RSL3+CHIR‑99021 group at the endpoint. Scan bar: **A** 50 μm. Statistical analysis: **B**, **D**, **F**, **H** Kruskal–Wallis test with Dunn’s post hoc test; data are presented as median with interquartile range (IQR); Significance levels: ns not significant; *P < 0.05; **P < 0.01; ***P < 0.001.

To further examine the functional relationship among FABP2, Wnt/β-catenin signaling, and GPX4, rescue experiments were performed in vitro and in vivo. In colon cancer cells with FABP2 knockdown, treatment with the GPX4 inhibitor RSL3 attenuated the enhanced proliferative, migratory, and invasive phenotypes. Subsequent activation of Wnt signaling did not further increase these effects (Fig. 6A–F). These findings are consistent with a model in which GPX4 functions downstream of Wnt/β-catenin signaling in FABP2-mediated ferroptosis regulation.

In vivo, pharmacological inhibition of Wnt signaling or GPX4 similarly reduced the increased liver metastatic burden induced by FABP2 knockdown. Consistent with the in vitro observations, activation of Wnt signaling failed to restore the enhanced metastatic phenotype when GPX4 activity was inhibited by RSL3 (Fig. 6G, H).

Together, these findings support the involvement of the Wnt/β-catenin/GPX4 signaling axis in FABP2-associated regulation of ferroptosis sensitivity and liver metastasis in colon cancer.

## Discussion

**Fig. 7:**
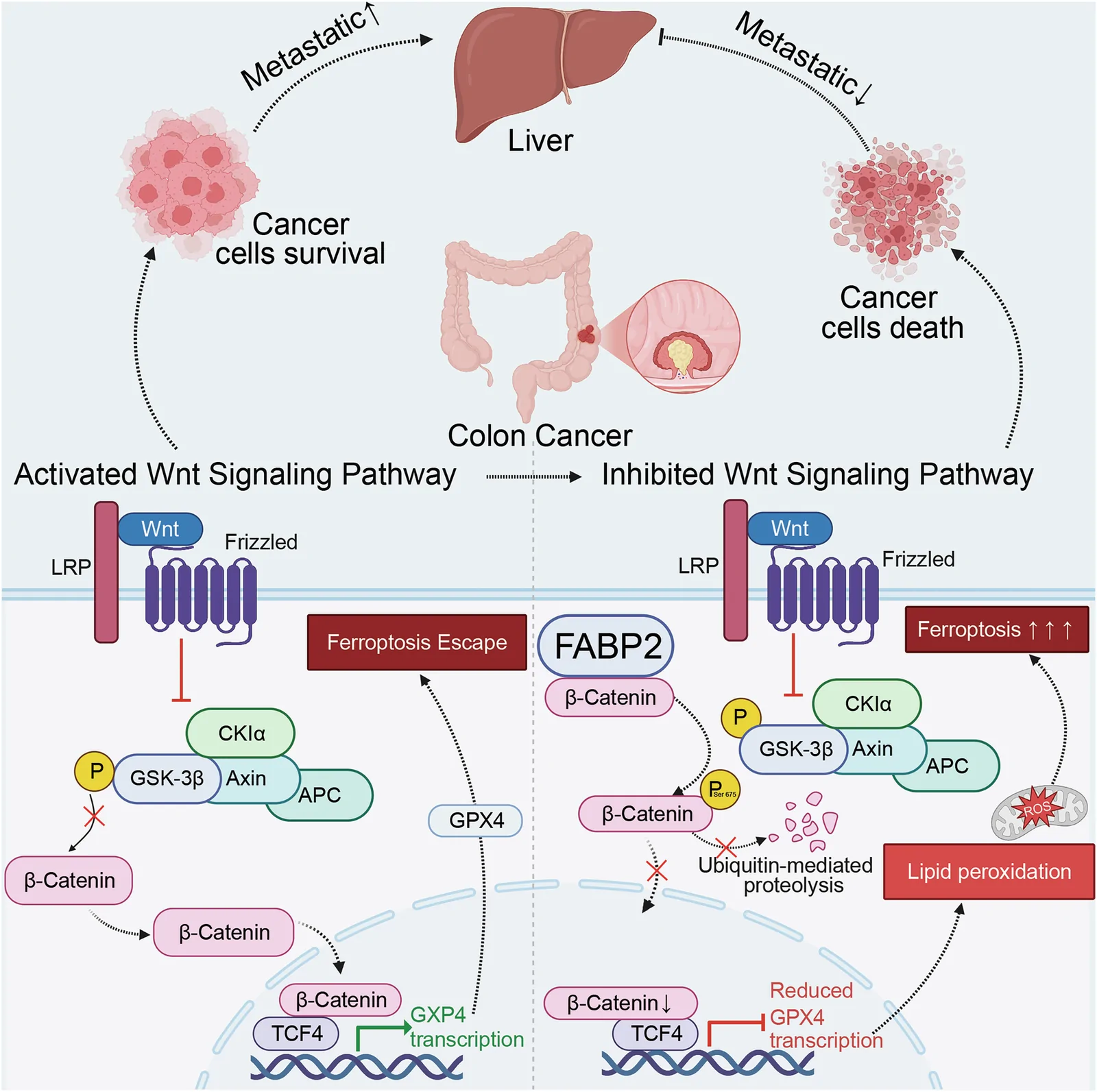
The mechanisms of FABP2 regulating liver metastasis in colon cancer (by Figdraw).

Colon cancer remains one of the leading causes of cancer-related morbidity and mortality worldwide, with liver metastasis representing a major determinant of poor clinical outcome [1, 39]. Although advances in surgical techniques and systemic therapies have improved disease management, effective strategies targeting the molecular mechanisms underlying metastatic colonization and treatment resistance remain limited [40, 41]. In the present study, we identified a FABP2-Wnt/β-catenin-GPX4 signaling axis associated with ferroptosis regulation and liver metastatic progression in colon cancer (Fig. 7).

Unlike canonical negative regulators of Wnt signaling, such as *APC* and *AXIN*, which limit β-catenin activity primarily through degradation [13], our findings suggest that FABP2 modulates β-catenin signaling through altered subcellular localization. FABP2 interacted with β-catenin, increased its cytoplasmic retention, and reduced nuclear accumulation despite elevated total β-catenin levels. This pattern may be particularly relevant in colon cancer, where APC mutations frequently impair canonical β-catenin turnover. Under these conditions, restriction of β-catenin nuclear accumulation may represent an alternative mechanism influencing downstream transcriptional activity.

Members of the FABP family have increasingly been implicated in tumor progression through regulation of lipid metabolism and intracellular signaling. For example, FABP4 has been reported to suppress migration, invasion, and epithelial–mesenchymal transition (EMT) in colon cancer cells, whereas FABP5 participates in lipid metabolic regulation and signaling pathways linked to tumor progression [42, 43]. In contrast, the role of FABP2 in colon cancer has remained poorly defined, particularly in the context of liver metastasis. In the present study, FABP2 expression was reduced in primary colon cancer tissues and was associated with tumor stage, metastatic progression, and poorer progression-free survival. Transcriptomic analysis further identified FABP2 among the downregulated genes associated with ferroptosis-related pathways. Together, these findings support a potential role for FABP2 in linking lipid metabolism with ferroptosis-related regulation during colon cancer progression.

Given the association between FABP2 and metastatic phenotypes, we next explored its functional role in ferroptosis regulation. Ferroptosis resistance is increasingly recognized as an adaptive mechanism that enables metastatic tumor cells to survive in the oxidative and iron-rich liver microenvironment [44–46]. Among the known regulators of ferroptosis, GPX4 is a central determinant of lipid peroxide detoxification and has been implicated in colorectal cancer progression, metastasis, and therapeutic resistance [47–50]. Our findings indicate that FABP2 is associated with altered GPX4 expression and ferroptosis-related phenotypes in colon cancer. FABP2 knockdown reduced lipid peroxidation and promoted proliferative, migratory, and invasive phenotypes, whereas FABP2 overexpression produced the opposite effects. Pharmacological induction of ferroptosis with RSL3 partially attenuated the effects of FABP2 knockdown in vitro and reduced liver metastatic burden in vivo, supporting a functional contribution of ferroptosis to FABP2-associated phenotypes. Together, these observations support a model in which FABP2 contributes to ferroptosis sensitivity, at least in part, through regulation of GPX4 expression. Given the importance of ferroptosis resistance during metastatic colonization, dysregulation of this pathway may facilitate liver metastatic progression in FABP2-low tumors.

To further define the mechanism linking FABP2 to GPX4 regulation, we examined upstream transcriptional pathways that may mediate this effect. Because FABP2 lacks intrinsic transcriptional activity, we focused on known regulators of GPX4 expression and identified β-catenin as a potential mediator. β-catenin forms a transcriptional complex with TCF4 and has recently been implicated in ferroptosis resistance through transcriptional regulation of GPX4 [14, 51], providing a mechanistic basis for investigating this pathway in the context of FABP2. Our data support an interaction between FABP2 and β-catenin and indicate that FABP2 influences β-catenin intracellular localization. FABP2 overexpression increased total β-catenin abundance while reducing its nuclear accumulation, suggesting that FABP2 modulates β-catenin signaling through altered subcellular distribution rather than reduced protein expression. This observation may be particularly relevant in colon cancer, where *APC* mutations frequently disrupt canonical β-catenin degradation and permit persistent pathway activation.

An additional finding of this study was the increased phosphorylation of β-catenin at Ser675 following FABP2 overexpression. Previous studies have shown that phosphorylation at this residue can stabilize β-catenin and reduce ubiquitination-dependent degradation [52]. This may explain the increase in total β-catenin protein observed in FABP2-overexpressing cells despite reduced nuclear accumulation. Although GSK3β activity is an established regulator of β-catenin turnover, our findings suggest that FABP2-associated β-catenin phosphorylation may not be explained predominantly by canonical GSK3β-dependent regulation. Prior work has implicated protein kinase A (PKA) in β-catenin Ser675 phosphorylation [53], raising the possibility that additional kinase pathways contribute to this process. Functionally, FABP2 knockdown increased TCF4 occupancy at the GPX4 promoter and enhanced GPX4 transcriptional activity, whereas pharmacologic inhibition of Wnt signaling attenuated GPX4 upregulation and metastatic phenotypes associated with FABP2 loss. Together, these findings support a model in which FABP2 influences ferroptosis-related phenotypes, at least in part, through regulation of β-catenin/TCF4-dependent GPX4 transcription.

The potential clinical relevance of this study lies in two aspects. First, reduced FABP2 expression was associated with tumor progression and liver metastatic phenotypes, suggesting that FABP2 may have value as a biomarker for identifying patients at increased risk of metastatic disease. Future studies in larger clinical cohorts will be needed to determine whether FABP2 expression provides prognostic value beyond established clinicopathological indicators. Second, our findings raise the possibility that ferroptosis-related vulnerabilities may differ according to FABP2 status. FABP2-low tumors showed increased GPX4 expression and reduced ferroptosis sensitivity, suggesting that modulation of the Wnt/β-catenin-GPX4 pathway may represent a potential therapeutic strategy in this context. Previous studies have shown that ferroptosis induction or GPX4 inhibition can suppress tumor progression and enhance treatment responses in colorectal cancer models [54–56]. Whether FABP2 expression may help identify tumors more responsive to ferroptosis-targeted interventions warrants further investigation.

Several limitations of this study should be acknowledged. First, in vivo experiments were performed using cell line-based models, which may not fully capture the heterogeneity and clinical complexity of human colorectal cancer. Future validation using patient-derived xenograft models or clinically relevant preclinical systems would strengthen translational relevance. Second, the portal vein injection model primarily reflects hepatic colonization rather than the complete metastatic cascade and therefore does not fully reproduce spontaneous liver metastasis. Orthotopic models may provide additional insight into metastatic progression in future studies. Third, although multiple complementary approaches supported an interaction between FABP2 and β-catenin, the structural determinants of this interaction remain incompletely defined. Finally, because FABP2 functions as a lipid-binding protein, whether its ligand-binding status contributes to β-catenin regulation and ferroptosis-related signaling warrants further investigation.

## Conclusion

In summary, our study identifies FABP2 as a regulator of β-catenin subcellular localization in colon cancer. FABP2 restricts nuclear β-catenin accumulation and is associated with reduced GPX4 expression, enhanced ferroptosis sensitivity, and suppression of liver metastatic phenotypes. These findings link a differentiation-associated lipid-binding protein to ferroptosis-related tumor progression and provide additional insight into the regulatory interplay between lipid metabolism and ferroptosis in colon cancer.

## Supplementary information

Supplementary Material

## Data Availability

Survival curves for FABP2 in colon cancer were generated using the Kaplan–Meier plotter (http://kmplot.com/analysis/). Gene expression data for FABP2 in human cancer tissues were extracted from the TCGA (https://portal.gdc.cancer.gov/) and GEO (https://www.ncbi.nlm.nih.gov/geo/) databases. Differential gene expression analysis was performed using the Birmingham Cancer Data Analysis Portal from the University of Alabama (https://ualcan.path.uab.edu/). The datasets generated during and/or analyzed during the current study are not publicly available until completion of ongoing analyses but are available from the corresponding author on reasonable request.
