# Supplementary material for: Fatty acid-binding protein 2 suppresses liver metastasis by cytoplasmic sequestration of β-catenin to enhance ferroptosis sensitivity in colon cancer
Source: Cell Death Dis. 2026 Aug 1;17(1):921. doi: 10.1038/s41419-026-09161-7 (PMC13720062; doi:10.1038/s41419-026-09161-7)

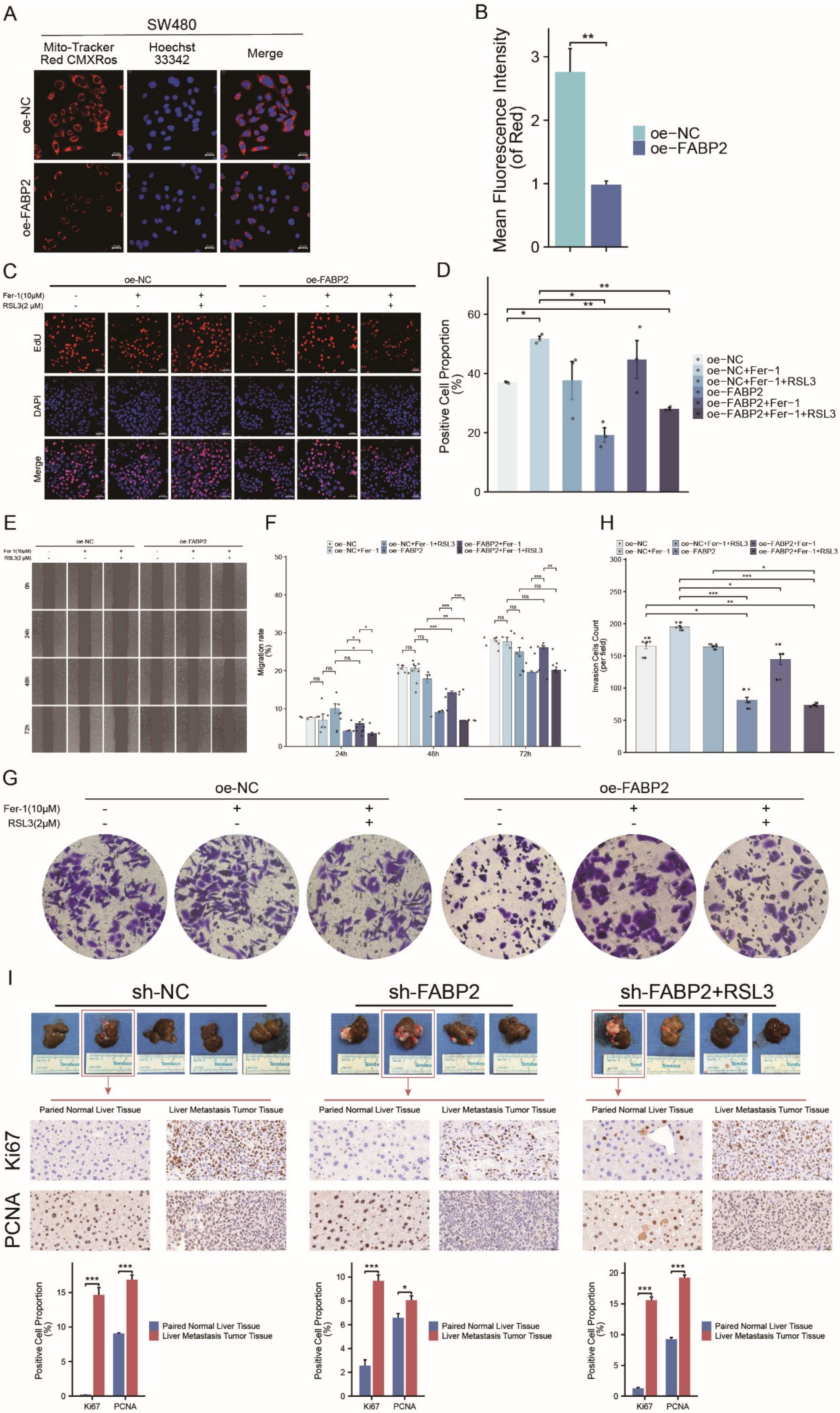

Supplementary Fig. S1 FABP2 regulates ferroptosis sensitivity and colon cancer

cell malignant phenotypes, related to Fig. 2.

(A-B) FABP2 overexpression enhances ferroptosis sensitivity. Representative fluorescence images of SW480 cells with FABP2 overexpression(oe-FABP2) or empty vector control (oe-NC) stained with Mito-Tracker Red CMXRos (mitochondrial membrane potential) and Hoechst 33342 (nuclei)(A). Quantification of mean Mito-Tracker red fluorescence intensity(B).

(C-H) Functional assays in SW480 cells with FABP2 overexpression. EdU proliferation assay(C-D), wound healing migration assay(E-F), Transwell invasion assay(G-H). Overexpression of FABP2 suppressed these malignant phenotypes, which were reversed by Fer-1 treatment and further inhibited by RSL3.

(I) Immunohistochemical analysis of liver metastatic lesions from mice in Figure 2N. FABP2 knockdown significantly increased proliferation marker positivity.

Scale bars: (A) 20 μm; (C) 50 μm.

Statistical analyses: (B, I) two-tailed t-test, data presented as mean ± SD; (D, F, H) Kruskal-Wallis test with Dunn's post hoc test, data presented as median with interquartile range (IQR); Significance levels: ns, not significant; *$P < 0.05$, **$P < 0.01$, ***$P < 0.001$.

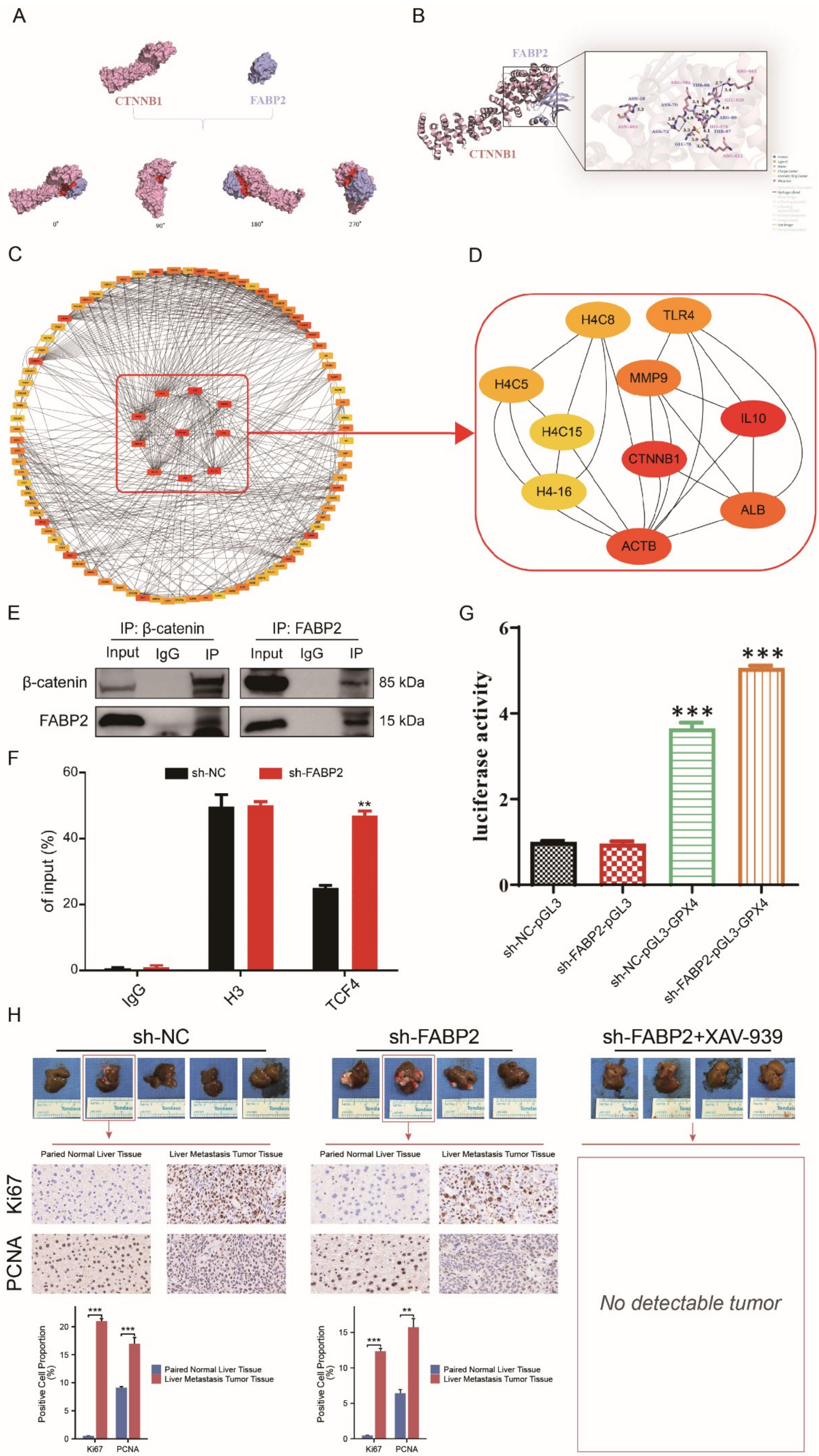

Supplementary Fig. S2 FABP2 interacts with β-catenin and regulates GPX4

transcription via the Wnt/β-catenin pathway, related to Figure 4.

(A-B) Protein-protein docking analysis predicted a potential interaction between FABP2 and CTNNB1 (β-catenin). Surface representation of docking result: CTNNB1 (pink), FABP2 (purple), and intermolecular contact surface (red)(A). Interaction between CTNNB1 (pink) and FABP2 (purple) identified by PLIP analysis(B).

(C-D) Transcriptome profiling identified CTNNB1 as a potential downstream effector of FABP2 in colon cancer cells. The top 100 differentially expressed genes ranked by statistical significance(C). The top 10 hub genes identified using Cytoscape software(D).

(E) Reciprocal co-immunoprecipitation (Co-IP) assays were performed in colon cancer cells using anti-β-catenin or anti-FABP2 antibodies, followed by immunoblotting for the indicated proteins. Co-IP analysis confirmed the association between FABP2 and β-catenin. IgG was used as a negative control.

(F) Chromatin immunoprecipitation-quantitative PCR (ChIP-qPCR) analysis showed that FABP2 knockdown significantly enhanced TCF4 occupancy at the GPX4 promoter region, indicating increased transcriptional regulation(n=3).

(G) Dual-luciferase reporter assays demonstrated that downregulation of FABP2 markedly increased the transcriptional activity of the GPX4 promoter.

(H) Representative images of H&E staining and Ki67 immunohistochemistry of paired normal liver tissues and liver metastatic tumor tissues from mice injected with sh-NC, sh-FABP2, or sh-FABP2 + XAV-939 cells. XAV-939 treatment partially reduced the metastatic burden and proliferative index (Ki67) induced by FABP2 knockdown.

Scale bars: (H) 20 μm.

Statistical analyses: (F, G) two-tailed t-test, data are presented as mean ± SD; significance levels: $^{**}P < 0.01$, $^{***}P < 0.001$.

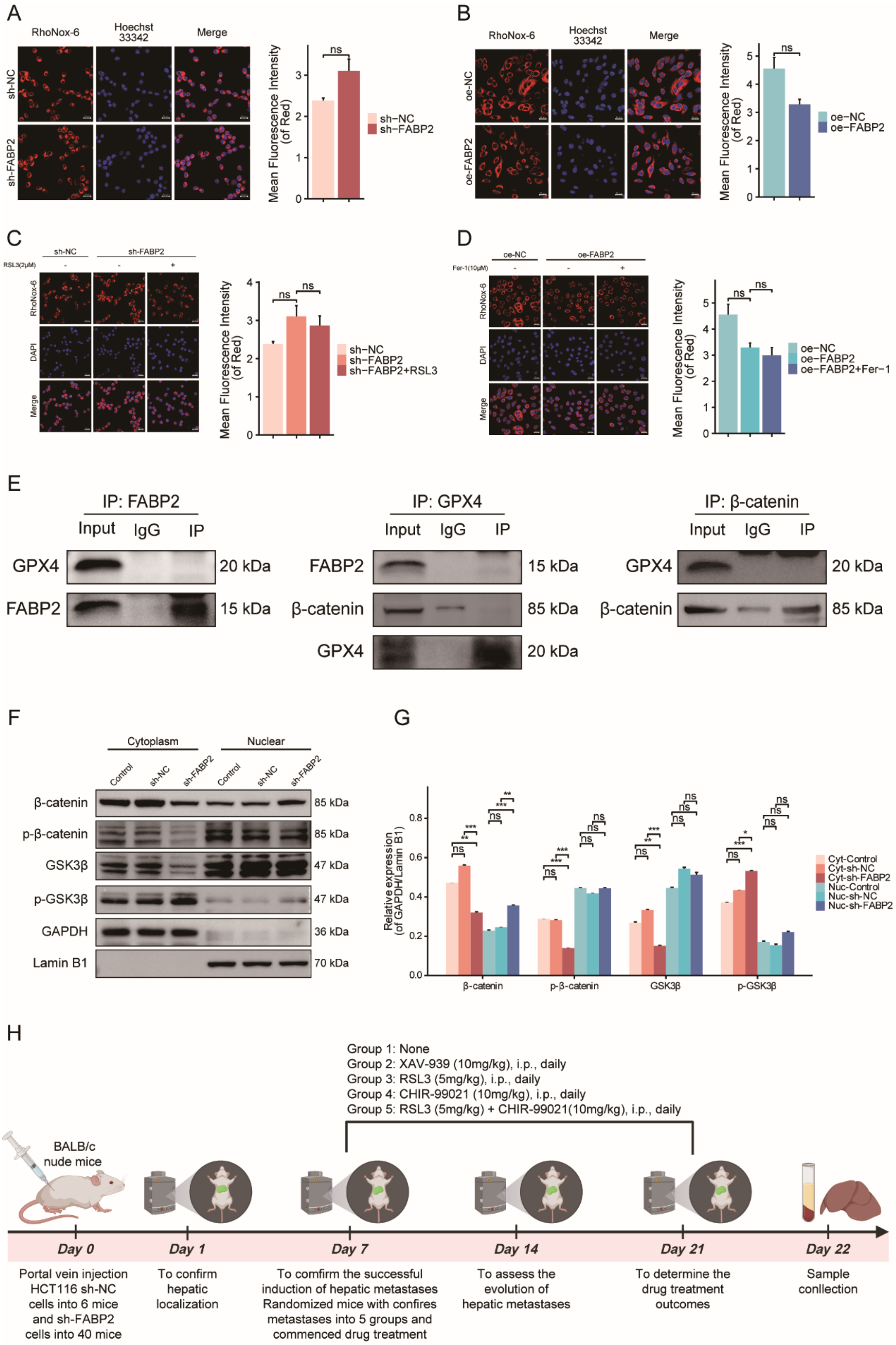

Supplementary Fig. S3 Regulation of ferroptosis by FABP2 is independent of Fe^{2+} levels or direct GPX4 binding but involves modulation of the β-catenin pathway. **(A-B)** Intracellular Fe^{2+} levels were not significantly altered by either FABP2

knockdown or overexpression.

(C-D) Treatment of FABP2 knockdown or overexpressing cells with the GPX4 inhibitor RSL3 or the ferroptosis inhibitor Fer-1 did not markedly change intracellular Fe^{2+} levels.

(E) Co-immunoprecipitation (Co-IP) analysis failed to detect direct interactions between GPX4 and FABP2 or β-catenin.

(F-G) Western blot analysis of phosphorylated β-catenin (p-β-catenin Ser675) in nuclear and cytoplasmic fractions, showing that FABP2 knockdown suppresses cytoplasmic β-catenin phosphorylation.

(H) Schematic timeline of the animal experiments.

Scan bar: (A-D) 20 μm.

Statistical analyses: (A-D) two-tailed t-test, data are presented as mean ± SD; (G) Kruskal-Wallis test followed by Dunn's post hoc test, data shown as median with interquartile range (IQR); significance levels: ns, not significant, $^{*}P < 0.05$, $^{**}P < 0.01$, $^{***}P < 0.001$.

Supplementary Table S1. Clinic Data Used in This Study.

No.	Test NO. (T/N)	Test Items	Sex	Age (year)	Diagnosis	Haight (cm)	Weight (Kg)	cTNM	Clinical Stage	Pathological type	pTNM	Pathological Stage	MLH1	MSH2	MSH6	PMS2	MSI	Her2	P53	Ki-67
1	1A/1a	RNA seq	Female	70	Ascending Colon Cancer	153	65	cT3N1 M0	IIIB	Moderately differentiated adenocarcinoma 60%, mucinous adenocarcinoma 40%	pT3N0 M0	IIA	+	+	+	+	MSS	2+	+	60-70%+
2	2B/2b	RNA seq	Female	66	Ascending Colon Cancer	148	53	cT4bN2 M0	IIIC	Moderately differentiated adenocarcinoma 70%, mucinous adenocarcinoma 30%	pT4aN1 cM0	IIIB	+	+	+	+	MSS	1+	+	90%+
3	3C/3c	RNA seq	Male	50	Sigmoid Colon Cancer	163	63	cT4aN2 M1b	IVB	Moderately differentiated adenocarcinoma 90%, mucinous adenocarcinoma 10%	pT4aN1 cM1b	IVB	+	+	+	+	MSS	2+	+	70%+
4	4D/4d	RNA seq	Male	59	Sigmoid Colon Cancer	166	45	cT4N0 M0	IIC	Moderately differentiated adenocarcinoma	pT4aN2 aM0	IIIC	+	+	+	+	MSS	0	+	90%+
5	5E/5e	RNA seq	Female	65	Sigmoid Colon Cancer	158	60	cT3N2 M0	IIIB	Mucinous adenocarcinoma	pT3N1b M0	IIIB	-	+	+	-	MSI-H	2+	+	80%+
6	T①/N①	IHC	Female	62	Ascending Colon Cancer	148	48	cT3N0 M0	IIA	Mucinous adenocarcinoma	pT2N0 M0	I	+	+	+	+	MSS	0	+	80%+
7	T②/N②	IHC	Female	74	Sigmoid Colon Cancer	140	45	cT2N1 M0	IIIA	Moderately differentiated adenocarcinoma	pT2N0 M0	I	+	+	+	+	MSS	0	-	90%+

Supplementary Table S2. List of Reagents and Antibodies Used in This Study.

Name	Category	Catalog Number	Source	Country/Region	Concentration/ ratio used	Application
RSL3	reagent	B6095	APExBIO	USA	In vitro, 2 µM<br>In vivo, 5 mg/kg	In vitro and vivo experiments
CHIR-99021	reagent	A3011	APExBIO	USA	In vitro, 10 µM<br>In vivo, 10 mg/kg	In vitro and vivo experiments
XAV-939	reagent	A1877	APExBIO	USA	In vitro, 5 µM<br>In vivo, 10 mg/kg	In vitro and vivo experiments
Fer-1	reagent	A4371	APExBIO	USA	10 µM	In vitro experiments
DMSO	reagent	GC203006	Servicebio	China	In vitro, <1‰<br>In vivo, 2%	In vitro and vivo experiments
D-Luciferin potassium salt	reagent	C3654	APExBIO	USA	15 mg/kg	In vivo experiments
FABP2 antibody	antibody	GTX83361	GeneTex	USA	WB, 1:1000<br>IHC, 1:800<br>IF, 1:800	WB, IHC, IF
FABP2 antibody	antibody	BY2571	Abways	China	1:50	Co-IP
GPX4 antibody	antibody	67763-1-Ig	Proteintech	China	WB, 1:2000<br>Co-IP, 1:50	WB, Co-IP
β-catenin antibody	antibody	66379-1-Ig	Proteintech	China	WB, 1:10000<br>IF, 1:800<br>Co-IP, 1:2000	WB, IF, Co-IP
TCF4 antibody	antibody	R389292	ZEN-BIOSCIENCE	China	1:50	Ch-IP
Phospho-beta Catenin (Ser552) antibody	antibody	AF8340	Afinity	China	1:2000	WB
Phospho-beta Catenin (Ser675) antibody	antibody	80084-1-RR	Proteintech	China	1:10000	WB
GSK-3β antibody	antibody	340449	ZEN-BIOSCIENCE	China	1:800	WB
Phospho-GSK3β (Ser9) antibody	antibody	R229943	ZEN-BIOSCIENCE	China	1:800	WB
FTH1 antibody	antibody	R23306	ZEN-BIOSCIENCE	China	1:5000	WB
ACSL4 antibody	antibody	22401-1-AP	Proteintech	China	1:5000	WB
SLC7A11 antibody	antibody	26864-1-AP	Proteintech	China	1:1000	WB
Lamin B1	antibody	P60054	Abmart	China	1:5000	WB
GAPDH	antibody	ZB15004-HRP	Servicebio	China	1:5000	WB

Abbreviations: WB, Western blot; IHC, Immunohistochemistry; IF, Immunofluorescence; Co-IP, Co-immunoprecipitation; Ch-IP, Chromatin immunoprecipitation; DMSO, Dimethyl sulfoxide; Fer-1, Ferrostatin-1; GPX4, Glutathione peroxidase 4; TCF4, Transcription factor 4; GSK-3β, Glycogen synthase kinase 3 beta; FTH1, Ferritin heavy chain 1; ACSL4, Acyl-CoA synthetase long-chain family member 4; SLC7A11, Solute carrier family

7 member 11; GAPDH, Glyceraldehyde-3-phosphate dehydrogenase.

Certificate of Analysis (COA) of human GPX4

Production Information			
**Gene Name**	Human GPX4	**Order No.**	G0365206-1
**Lot No.**	20250527/40-8-YLB40820	**Cloning Vector**	pGL3-Basic
**Cloning Sites**	SacI(GAGCTC)-HindIII(AAGCTT)	**Insert Size**	1517bp
**Competence**	Stbl3	**Vector Resistance**	Ampicillin

QC Results		
**Test Items**	**Specifications**	**Results**
**Insert Sequence**	Insert sequence results consistent with target	Pass
**Vector Sequence**	Flanking sequence consistent with expected	Pass
**ORF Across Junction**	Correct and consistent with target	N/A
**Restriction Digest**	Expected fragment sizes observed	Pass
**PCR Amplification**	Correct without non - specific bands	N/A
**DNA Quantity/Quality**	Concentration (n/a if lyophilized)	N/A
	Purity (A 260/A280 =1.8 - 2.0)	Pass
	Matrix	TE (lyophilized)
**Endotoxin Test**	Verified, <0.1 EU/µg (Endo-Free Preps Only)	N/A
**Appearance**	Clear, no visible particles	Pass
**Label**	Correct and white	Pass
**Comments**	-	-

Restriction Digestion Map

**Marker**	**1 2 M**	**Description of Map**
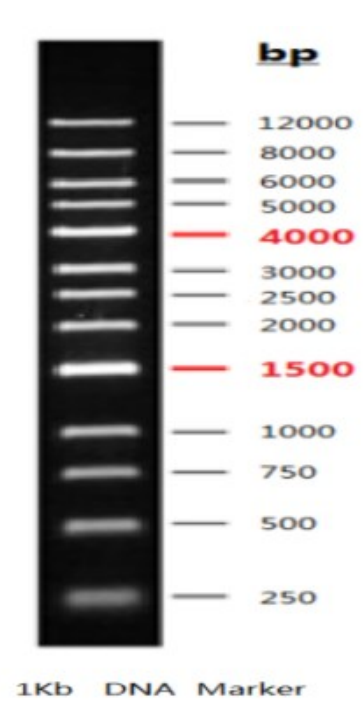	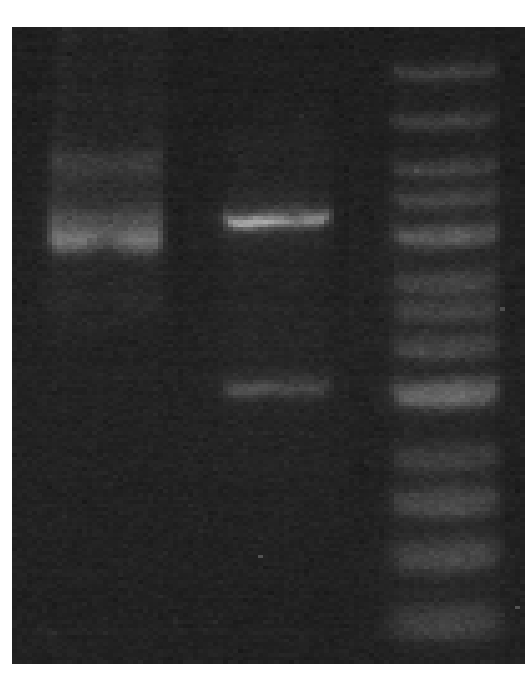	**Lane 1:** Plasmid<br>**Lane 2**: Plasmid Digested with SacI-HindIII<br>**Lane M**: DNA Marker

Certified by:<u>**HYC**</u>
Date:<u>**5-28-2025**</u>

TCF4 binding information to the GPX4 promoter region

GPX4 promoter sequence (yellow: binding region, red: transcription start site, blue: primer binding region):

>ref|NC_000019.10|:1102506-1104138 Homo sapiens chromosome 19, GRCh38.p14 Primary Assembly

GGCAGGAGAATCACTTGAACCCGGAAAGCGGAGGTTGCAGTGAGCTGCGATGGA
GCCACTGCACTCCAGCCTGGGTGACAGACCAAGACTCCTCAAAAAAAAAAAAAA
AAATCCAAACCCCTGCCTGTACAGGGGTCAAAGTCCAATCACGGTGACTCAGTTC
CCAAGTATGAGATGAGTGTTTCTGTTGCCACATGTCCAAGCCACGAAGTGAAAAA
CAGAGTTGGAAGCCAAGATGTGGGGCAGTATGGGCTCGGGTGTGCCCATACCTTG
TAATGTTAGAAGTGTAAGAAAACCCTCCAGACTTGTGTTTATTTTCTGAGCACAT
ACCGCGCGTCCCTGCGTGGGCAGCTGCCCCTGGGGGTGGGAGCCCAGGAAACTC
CAGGTTCTCAGTTAAGTGGTAACTGTCTTGATGACTCTCAGGTCTCAGTGCCCACA
TTATACAAGCGGCTCAGCAACCAGACCCAGGATGGGGAGTCACTGACCCCCTGC
GCCCATGGCAGGGGTGAGGGTAGGGACACCCCCATAAGATGGAGCTGTGATGAG
AAGCAAACCCAACTCAGTTTGGTTTCATTTACATTAAAAAGGCAAATCCCTTGGC
CGGATGCGGTGGCTCACACCTGTAATCCCAGCACTCTGGGAGGCCTAGGCGGGCA
GATCACCTGAGGTCAGGAGTTCGAGATCAGCCTGGCCAACATGGTGAAACCCCGT
CTCTACTAAAAATACAAAAAAAATTAGCTGGGTGTGGTGGCTCGCTCGTGTAATCC
CAGCTACTCAGGAGGCTGAGTCAGGAGAATCGCTTGAACCCGGGGGCTGGGTGG
AGGTTGCAGTGAGCTGAGATAGCGCCACTGCACTCCAGCCTGGGCAAAAGAGCA
AAAAAACTGTGTCTCGGAAAAAAAAAAAAAAAAAAGGCAAATCCCGGCCGAGC
CTTTGGCTCACGCATGTGATCCCAGCACTTTTGGGAAGCTGAGGCTGGAGGATCA
CTTGAGGTCAGAAGTTTGAGACCAGCCTAGGCAACATAGCGAGACCCCCATGTCT
ACAAAACAATAATTAGCCAGGAATGGTGGATGAGCCTGTTGTCCCAGCTACTCGG
GAAGCAGAGACGGGAGGTTCGCTTGAGCCTGGGAGGTCGAGGCTGCAGTGTGCC
CTGATCGTGCCACTGCACGTTAGCCTGGGCAACACAGCAAGACCCCGTGTCTTAA
AAAAAAAAAGGTAAATAAGAGTTGGGGACACTTTTCTGCGAGTTGGAGAAACCA
AACCCCCTCGACGAGTCTTAAGTAGTATTCTCAGGTTGTCTGGCCTGCGGCCGCG
CTGCTGTCAACCAGCCGGATAACTGCGCTGCCTCCCGACGCGCCGCCAGCCAGGC
CACGGCCTCCTAGACACAAGCGAGCATGCGCAGTCGCCAACAACAAGTCCGCAC
GTCCGGTCCCGCCCCCCCTTCCCCGCCTTCTTCCCACTCCGGCCTCCCATTGGCTG
ACGTCGGCGCGAGCGCTCAACACCGACGCG

Supplemental Material-Original Blots

Figure 1J

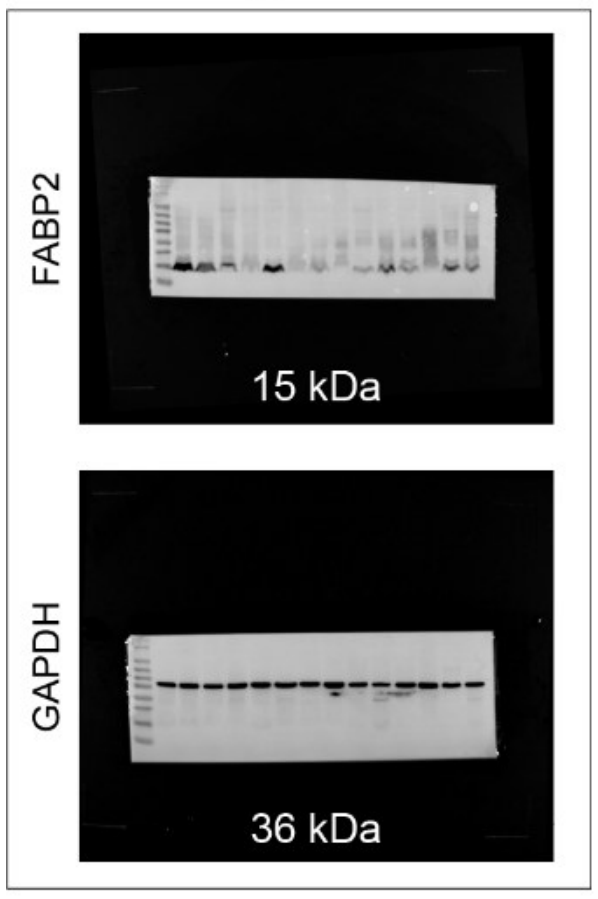

Figure 3C

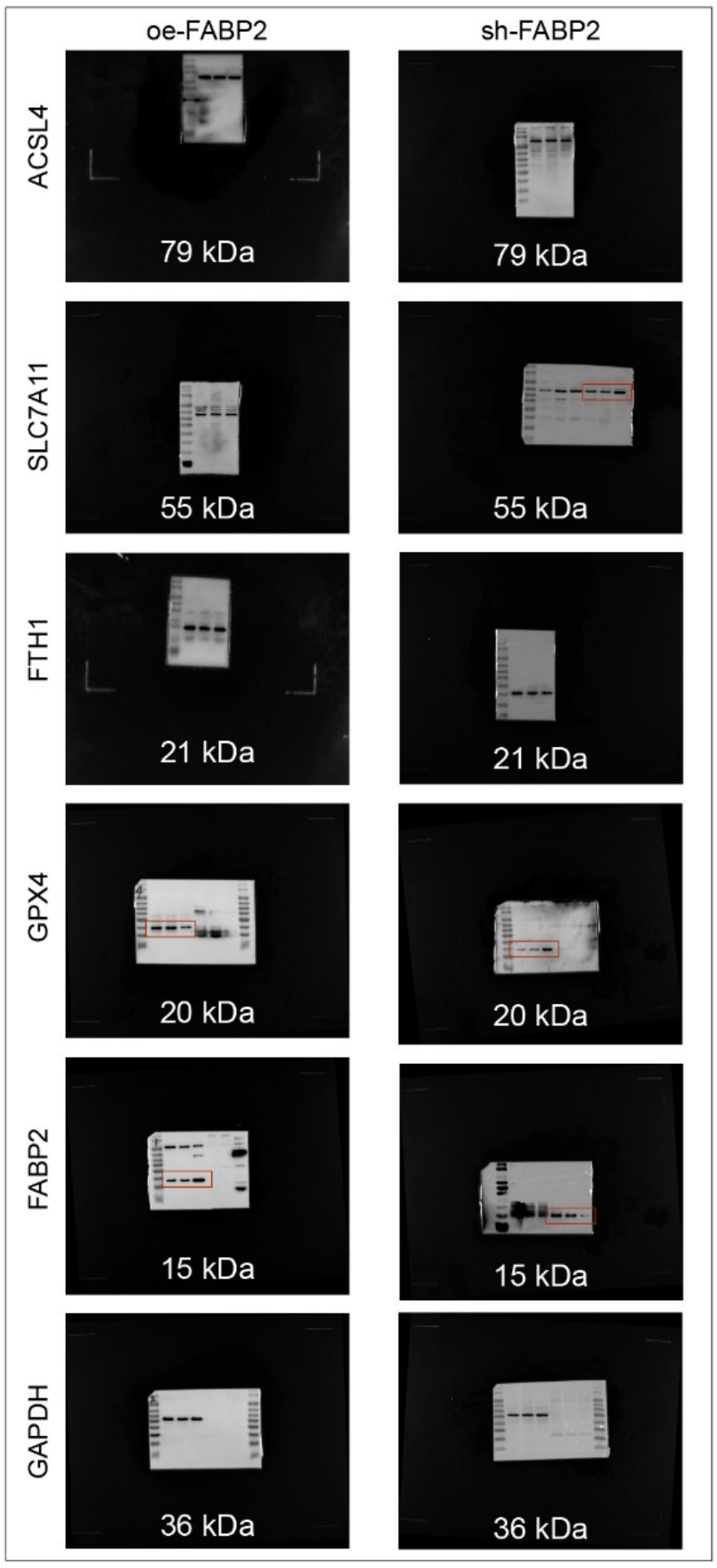

Figure 3G

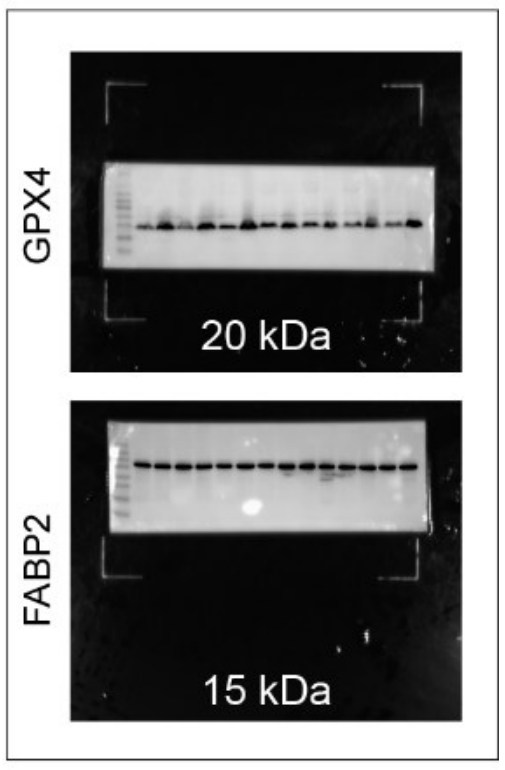

Marker

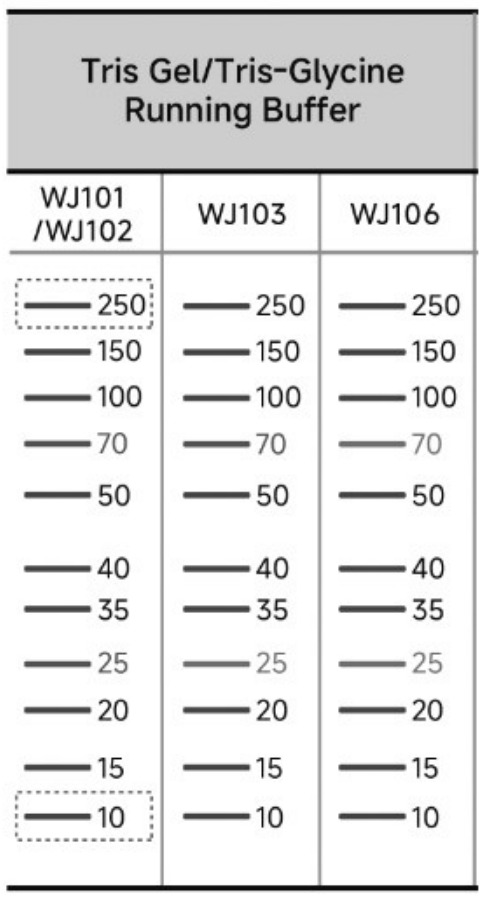

Figure 4G

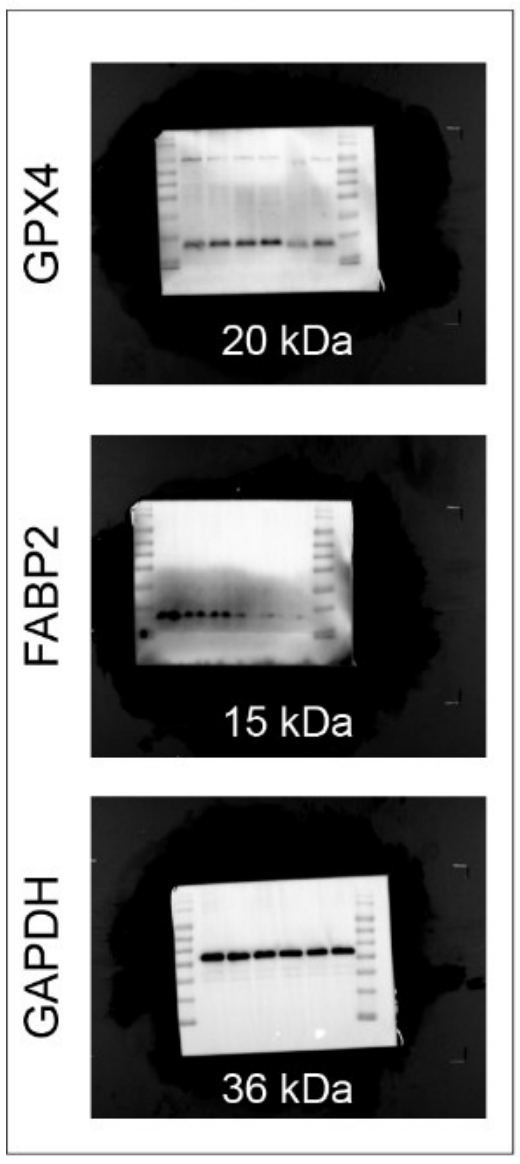

Figure 4I

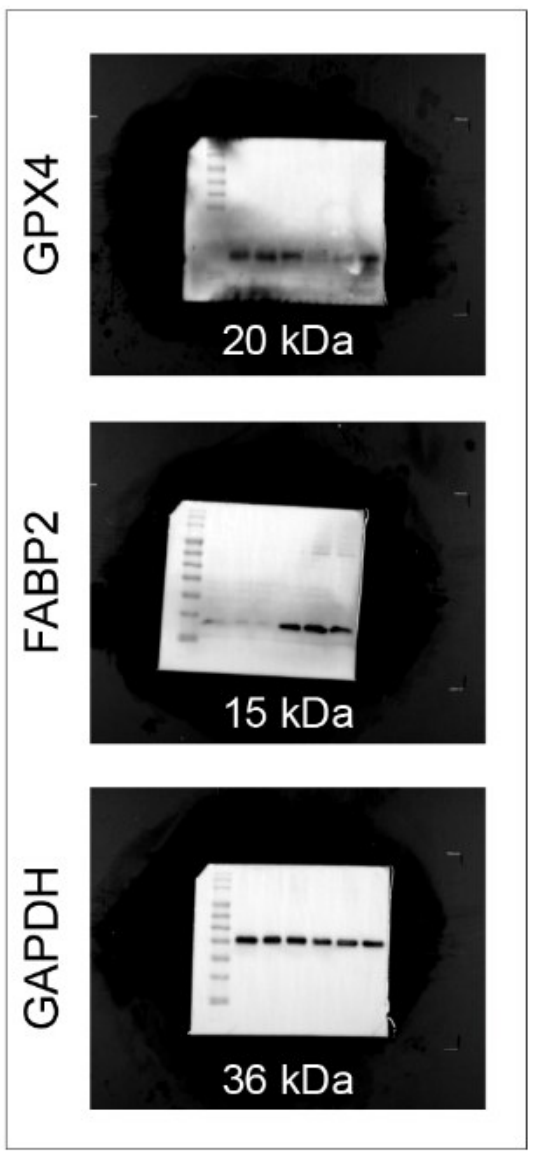

Figure 5A

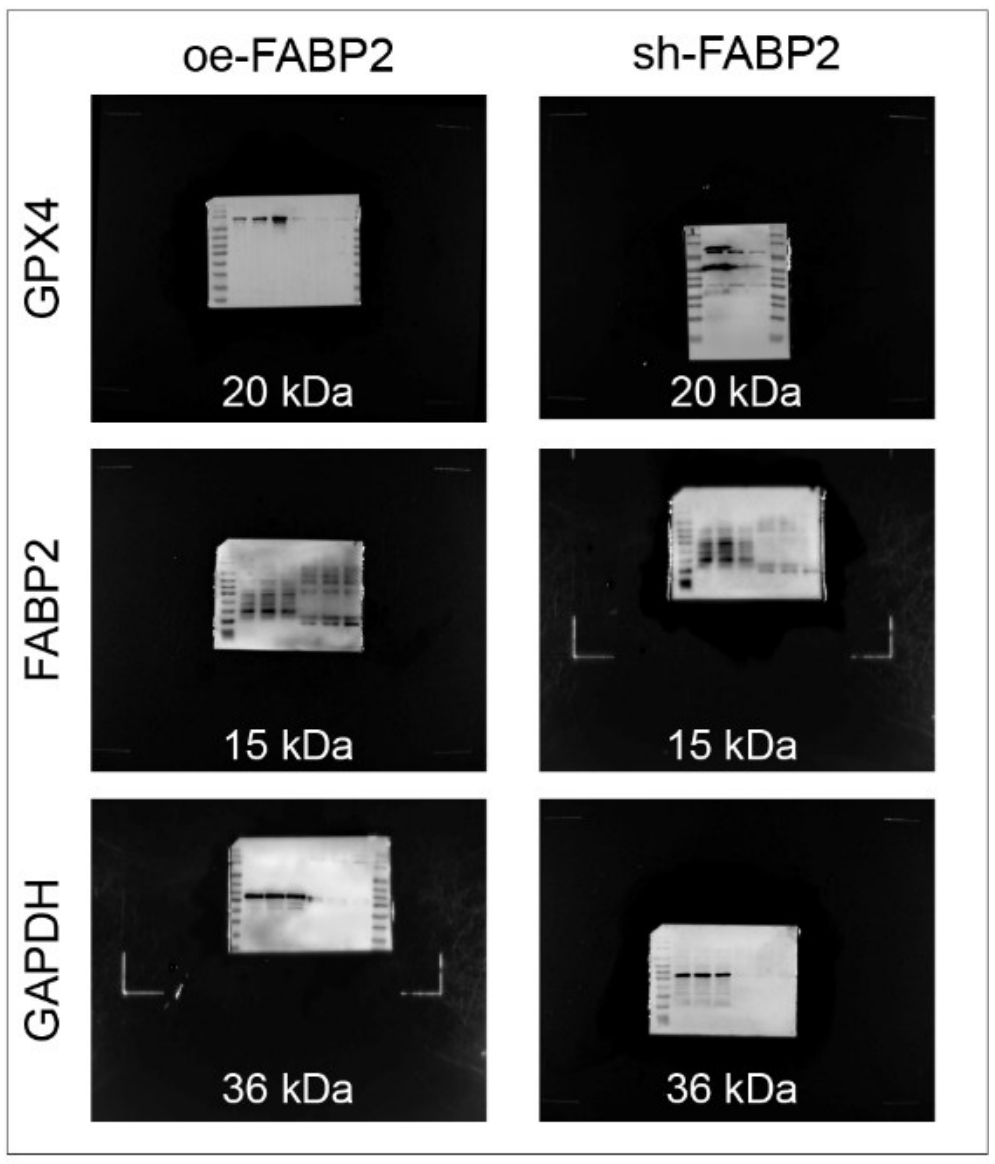

Marker

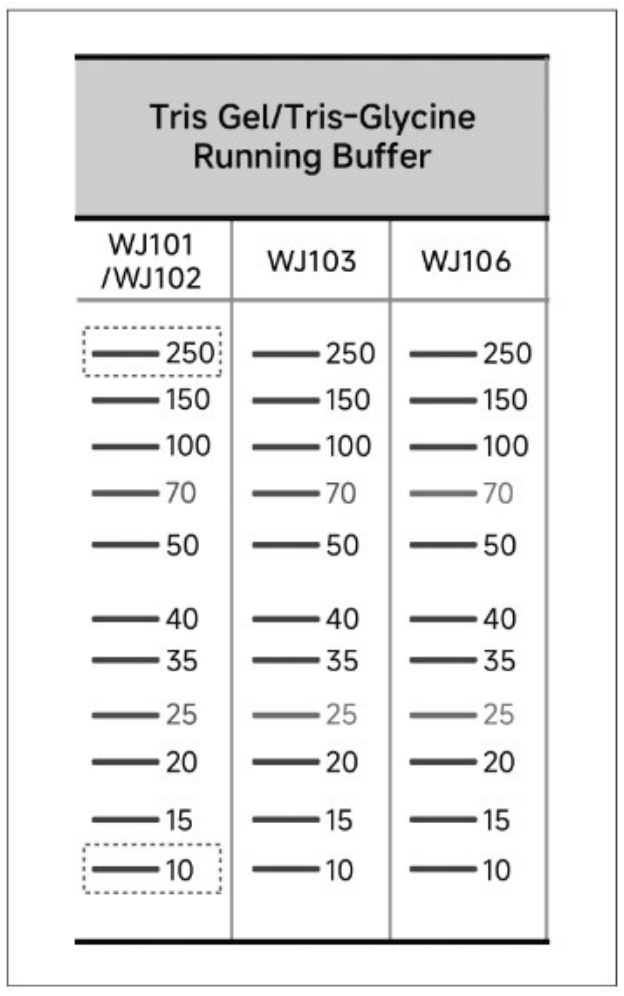

Figure 5C

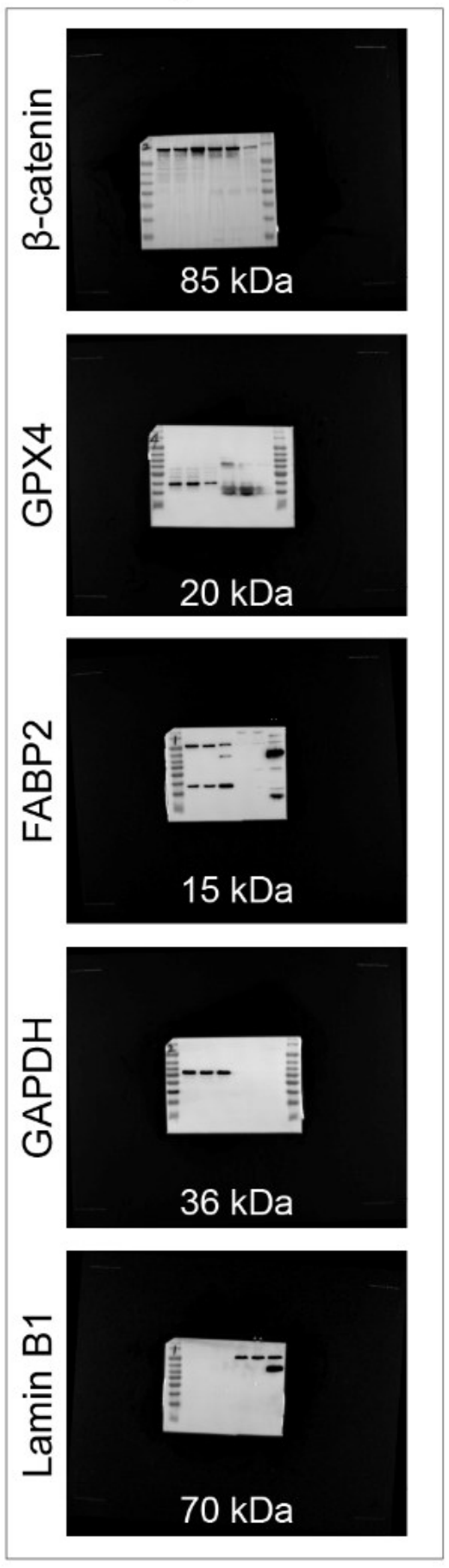

Figure 5K

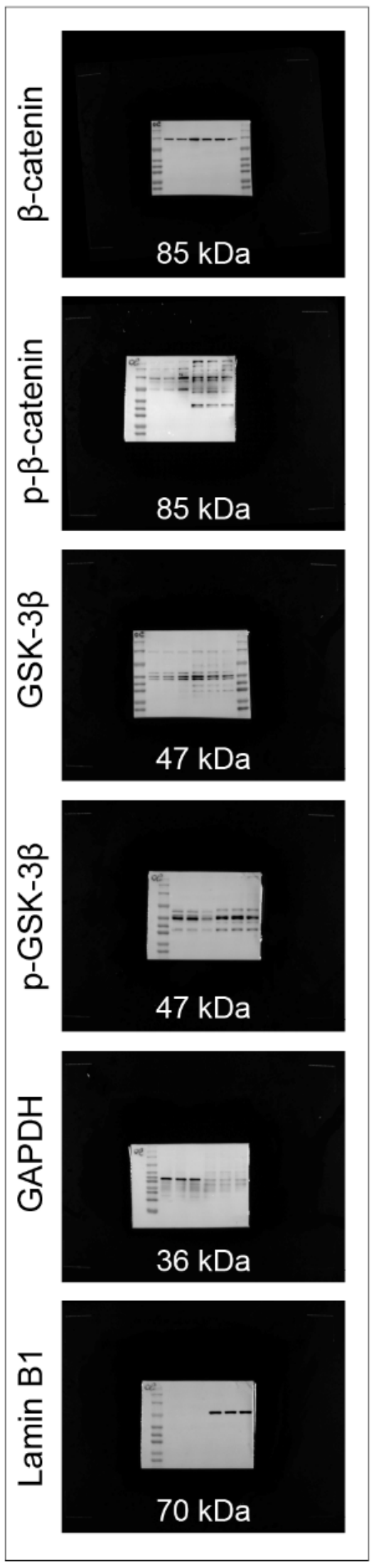

Figure S2E

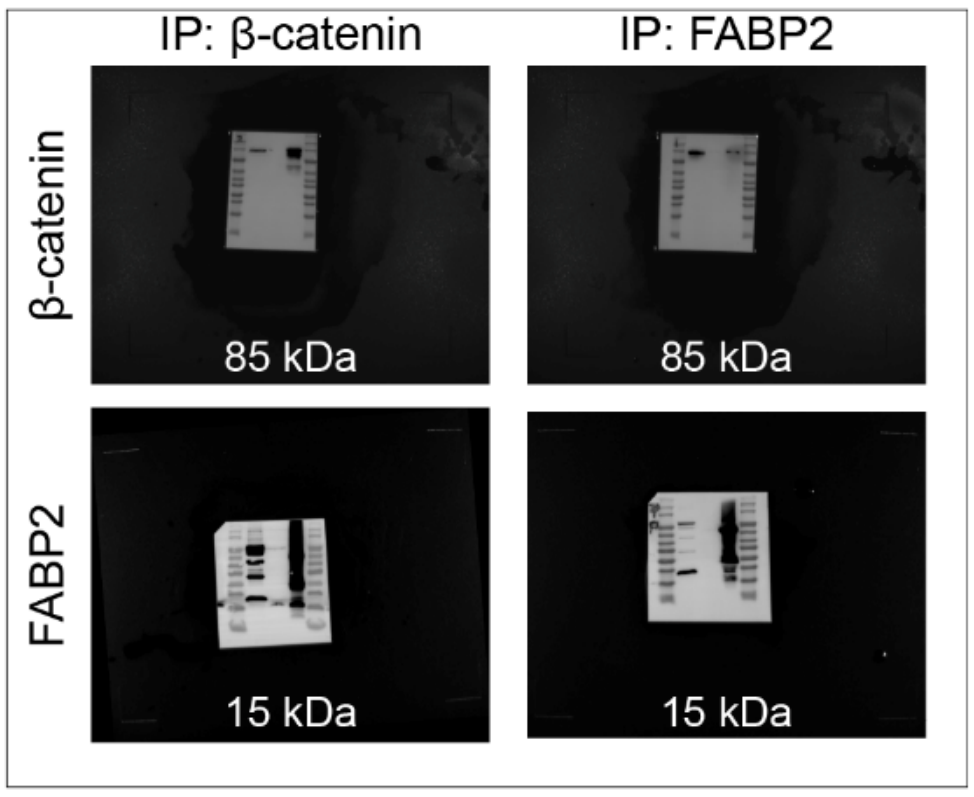

Figure S3E

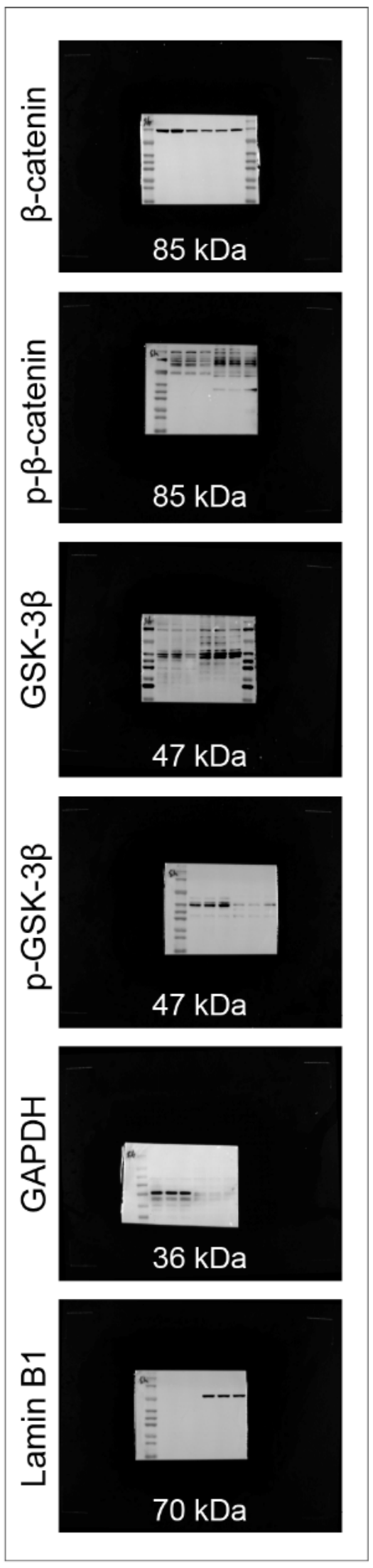

Marker

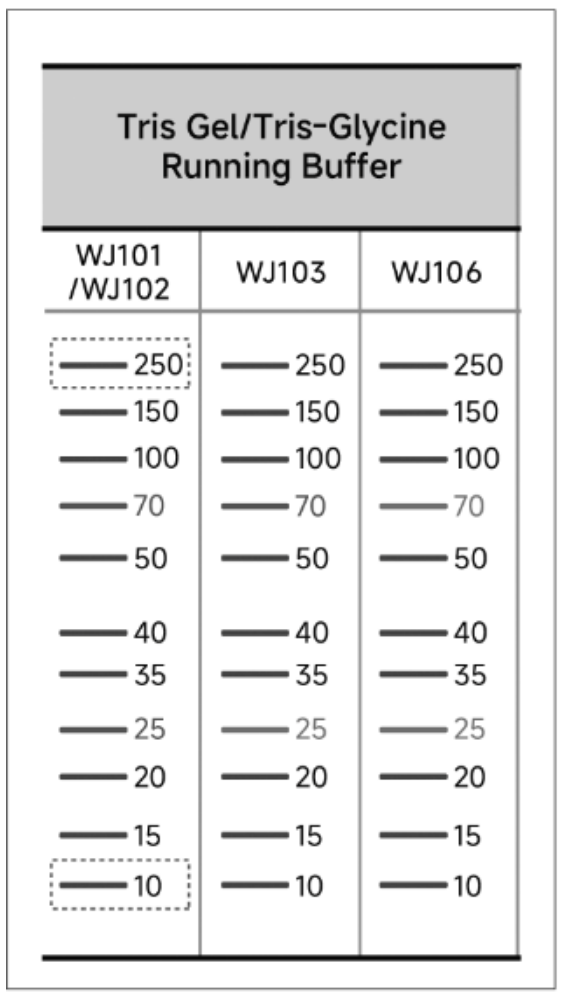

Figure S3F

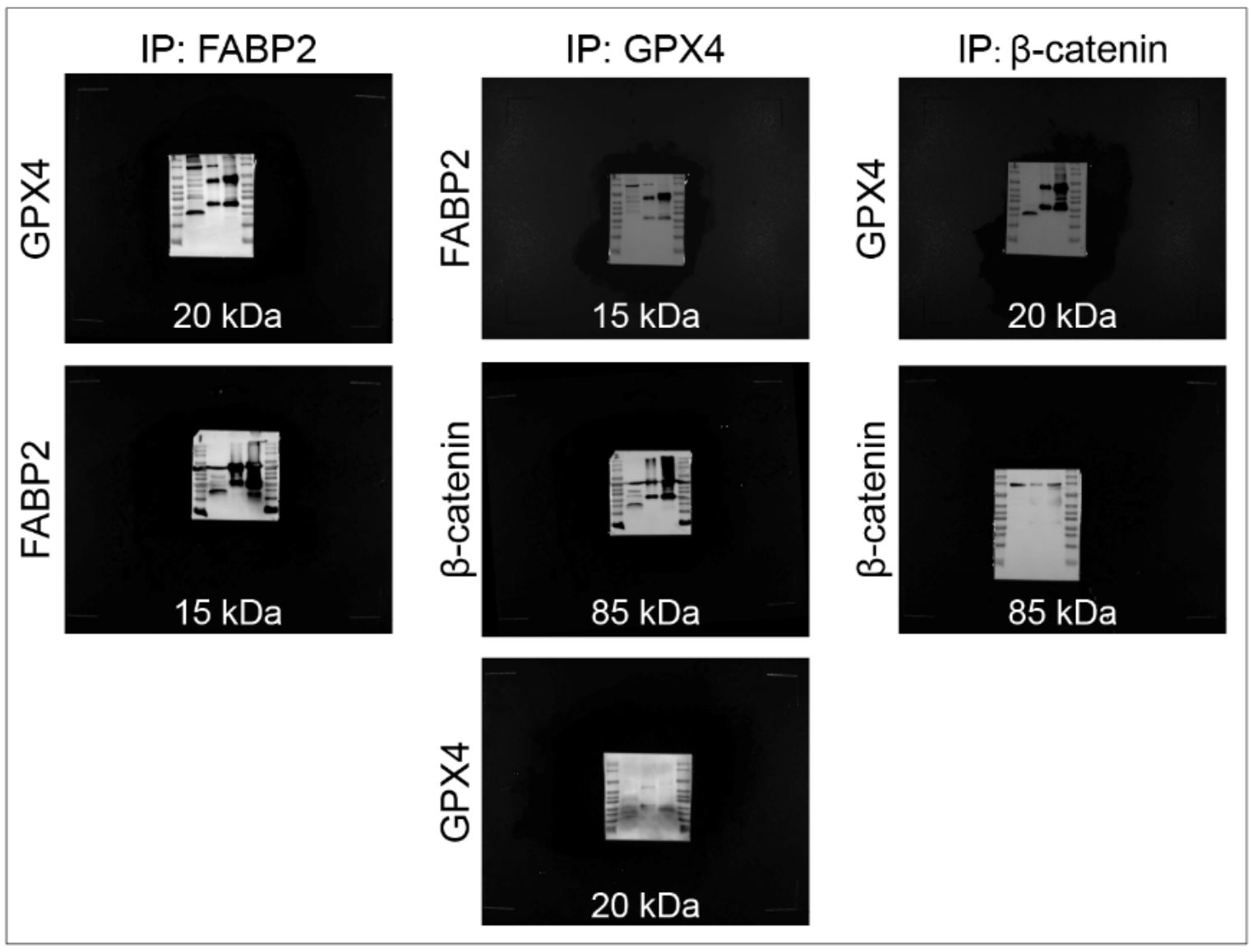

Supplement: Supplementary file 1 [file 41419_2026_9161_MOESM1_ESM.pdf]
